# Tautomerism and Self-Association in the Solution of New Pinene-Bipyridine and Pinene-Phenanthroline Derivatives

**DOI:** 10.3390/molecules25020298

**Published:** 2020-01-11

**Authors:** Atena B. Solea, Ivan Cornu, Vera Deneva, Aurelien Crochet, Katharina M. Fromm, Liudmil Antonov, Christophe Allemann, Olimpia Mamula

**Affiliations:** 1 Haute Ecole d’Ingénierie et d’Architecture Fribourg, HES-SO University of Applied Sciences of Western Switzerland, Pérolles 80, CH-1705 Fribourg, Switzerland; Atena-Bianca.Solea@hefr.ch (A.B.S.); ivan.cornu@master.hes-so.ch (I.C.); aurelien.crochet@unifr.ch (A.C.); Christophe.Allemann@hefr.ch (C.A.); 2 Department of Chemistry, University of Fribourg, Chemin du Musée 9, CH-1700 Fribourg, Switzerland; katharina.fromm@unifr.ch; 3 Bulgarian Academy of Sciences, Institute of Organic Chemistry with Centre of Phytochemistry, Acad. G. Bonchev street, bl. 9, 1113 Sofia, Bulgaria; vdeneva@orgchm.bas.bg (V.D.); Lantonov@orgchm.bas.bg (L.A.)

**Keywords:** tautomerism, pinene derivatives, chiral ligands, molecular spectroscopy, theoretical description, keto-enol tautomerism, H-bonding

## Abstract

Two novel pinene-type ligands have been synthesized and their tautomeric and self-associating behavior studied in solution and in the solid state. The first ligand, an acetylated derivative of 5,6-pinene-bipyridine, displays keto-enol tautomerism in solution. This tautomeric equilibrium was studied by NMR and UV-Vis spectroscopy in various solvents, and the results were compared with the ones obtained through DFT calculations. The second ligand was obtained by an unusual oxidation of the phenanthroline unit of a pinene-phenanthroline derivative. This compound exists as a single tautomer in solution and aggregates both in solution (as observed by NMR) and in the solid state through H-bonding as observed by X-ray structure determination and confirmed by DFT studies.

## 1. Introduction

Ligands with an annellated pinene backbone have been used extensively for chiral induction in coordination compounds of *d* and *f* metal ions, giving rise to well defined, enantiopure supramolecular structures [1,2,3,4,5]. Their ability to control the metal centers’ chirality makes them attractive not only in the field of supramolecular chemistry [2], but also for various applications like enantioselective catalysis or single chiral molecular magnets [6,7,8,9]. Among the pinene containing ligands, the most studied ones possess a bipyridine unit connected to the pinene moiety through either the 4,5 or 5,6 carbon atoms on the pyridine ring. These ligands can be obtained through a facile and scalable synthesis utilizing a Kröhnke salt as the intermediate [10]. An important and useful feature of these ligands is the facile deprotonation of the methylene unit close to the nitrogen atom of the pyridine ring. Moreover, due to steric effects, the deprotonation proceeds stereoselectively in the presence of strong bases like lithium diisopropylamide (LDA), rendering the diastereoselective functionalization of these ligands possible [11]. There are examples of pinene units functionalized with alkyl [12,13], carboxyl [11], or alcohol groups [14], among others. Another important class of ligands derived from pinene are the so-called CHIRAGEN (chiral generators) ligands, which have two bridged 5,6- or 4,5-pinene-bipyridine units [15,16]. These CHIRAGEN ligands can also predetermine the chirality of the metal centers [17,18,19]. 

The pinene-type ligands based on 1,10-phenantroline units [7,20,21] have been less studied than the ligands mentioned above, because their synthesis is more difficult and the rigidity of the phenanthroline unit narrows down their abilities to form discrete (supramolecular) species. Within the small family of 2,3-pinene-1,10-phenantroline ligands, so far only alkyl functionalization of the pinene unit has been reported to this date by Chelucci and co-workers [7,20,21].

Here, we report the synthesis of two new pinene-type ligands based on 2,2’-bipyridine and 1,10-phenanthroline units, as well as the study of their tautomeric behavior. The acetyl functionalized 5,6-pinene-bipyridine Compound **3** features a keto-enol tautomerism. The second ligand, **6**, obtained by the oxidation of the phenanthroline unit, exists in solution as a single tautomer, which forms dimers by H-bonding. The keto-enol and monomer-dimer equilibria have been investigated by variable temperature (VT) NMR and UV-Vis measurements and the results confirmed by DFT calculations and X-ray studies. To the best of our knowledge, this is the first tautomeric study of pinene containing compounds.

## 2. Results and Discussion

**Scheme 1:**
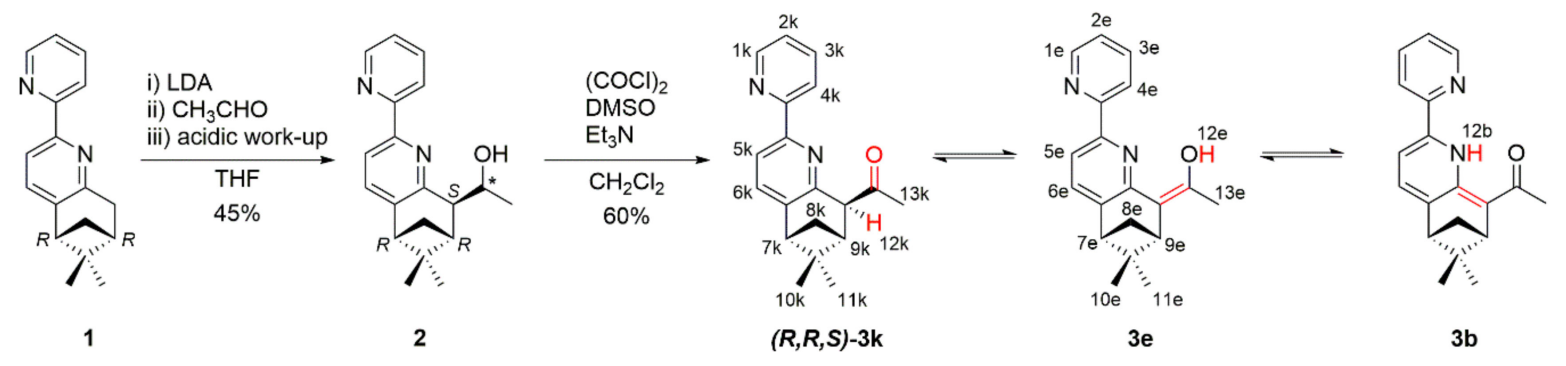
Synthesis of **3** and its tautomeric forms. LDA, lithium diisopropylamide.

The 5,6-pinene-bipyridine derivative **3** (Scheme 1) was obtained from the (−)-(R,R)-5,6-pinene-bipyridine **1**, which was synthetized as reported in the literature [10]. Deprotonation of **1** and reaction with acetaldehyde gave an *RRS*/*RRR* mixture of diastereomers (ratio 2:1) of Compound **2** (Figure S4). The preferred *S* configuration of the newly formed chiral carbon center was due to the steric hindrance of the pinene unit. Swern oxidation of **2** gave the desired Compound **3**. 

**Figure 1:**
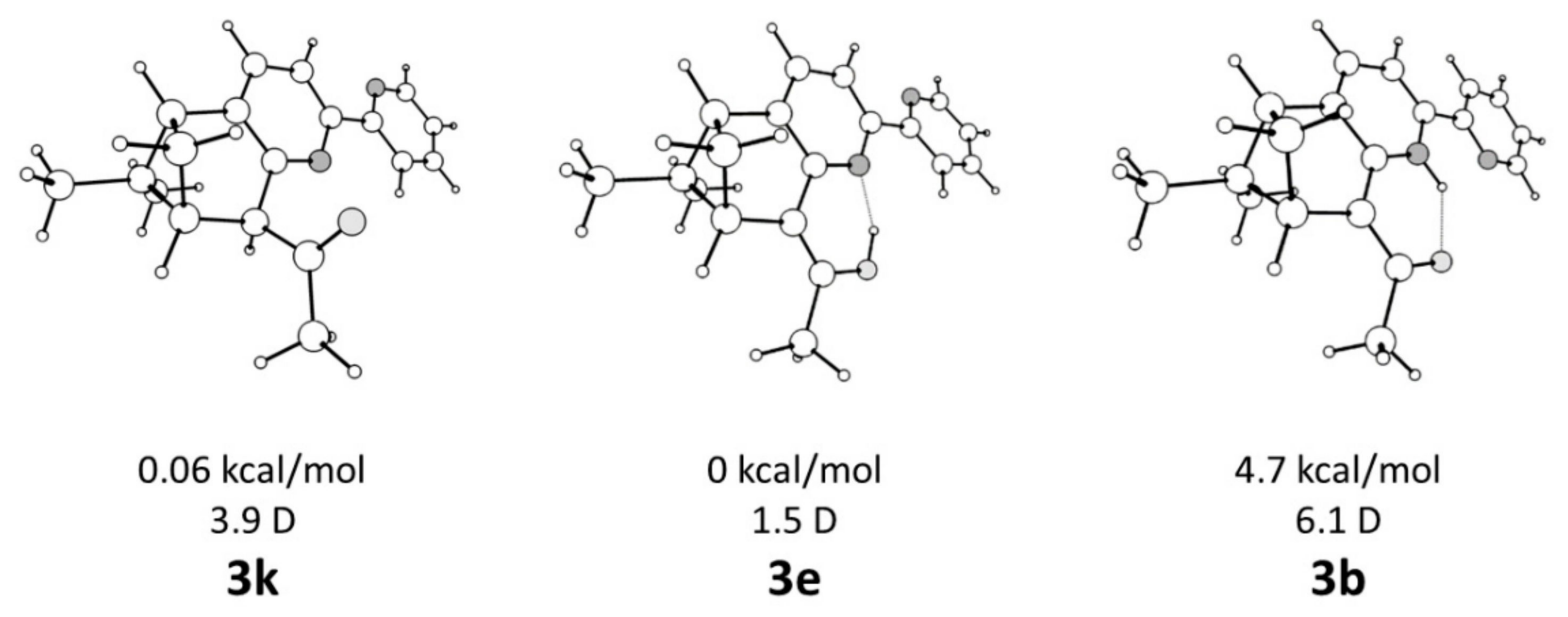
Optimized structures of the tautomers of **3k**, **3e**, and **3b** in CHCl_3_ with the relative energies to **3k** and their dipole moments.

The 5,6-pinene-bipyridine derivative **3** had three different tautomeric forms (keto, enol, and base NH, correspondingly **k**, **e**, and **b**) as shown in Scheme 1. According to the theoretical calculations (Figure 1), the keto and enol tautomers could co-exist in comparable amounts in CHCl_3_, CH_2_Cl_2_, and CH_3_CN. The corresponding theoretically predicted relative energies of the keto (**3k**) and enol (**3e**) tautomers are summarized in Table 1. A similar tautomeric preference has been observed by Gawinecki et al. [22] in 1-phenyl-2-(pyridin-2-yl)ethan-1-one, where structural similarity with **3** in the tautomeric backbone exists. The tautomer **3b** could not be observed in solution as its relative energy exceeded 4 kcal/mol. The stabilization of **3k** and **3e** over **3b** was not surprising. The expected additional stabilization in **3b** through NH…N-bonding was weak because a five membered cycle was formed, and the orientation of the nitrogen atoms led to steric hindrance between the C4 and C5 protons. The strength of the resonant assisted hydrogen bonding in **3e** and **3b**, estimated as the energy difference with corresponding anti CHO and COH isomers, was estimated as 8.7 and 6.7 kcal/mol, respectively. 

Theoretical calculations showed that **3k** and **3e** had almost the same energy. As a consequence, both tautomers should be detected in solution. UV-Vis spectra showed two major long wavelength bands in all solvents (Figure S3). The intensive composite band around 290 nm belonged to **3k** according to the theoretical calculations (two sub-bands at 290 and 285 nm with oscillator strengths of 0.54 and 0.11 resp. in acetonitrile) along with a red shifted shoulder at ~350 nm, attributed to **3e** (368 nm, oscillator strength 0.13). The different dipole moments of the tautomers suggested that the equilibrium was solvent dependent. Moreover, modifying the solvent caused light spectral changes that could be attributed to the substantial difference in the intensities of the bands and to the strong overlapping between them. This overlap did not allow a quantitative interpretation of the equilibrium. 

**Figure 2:**
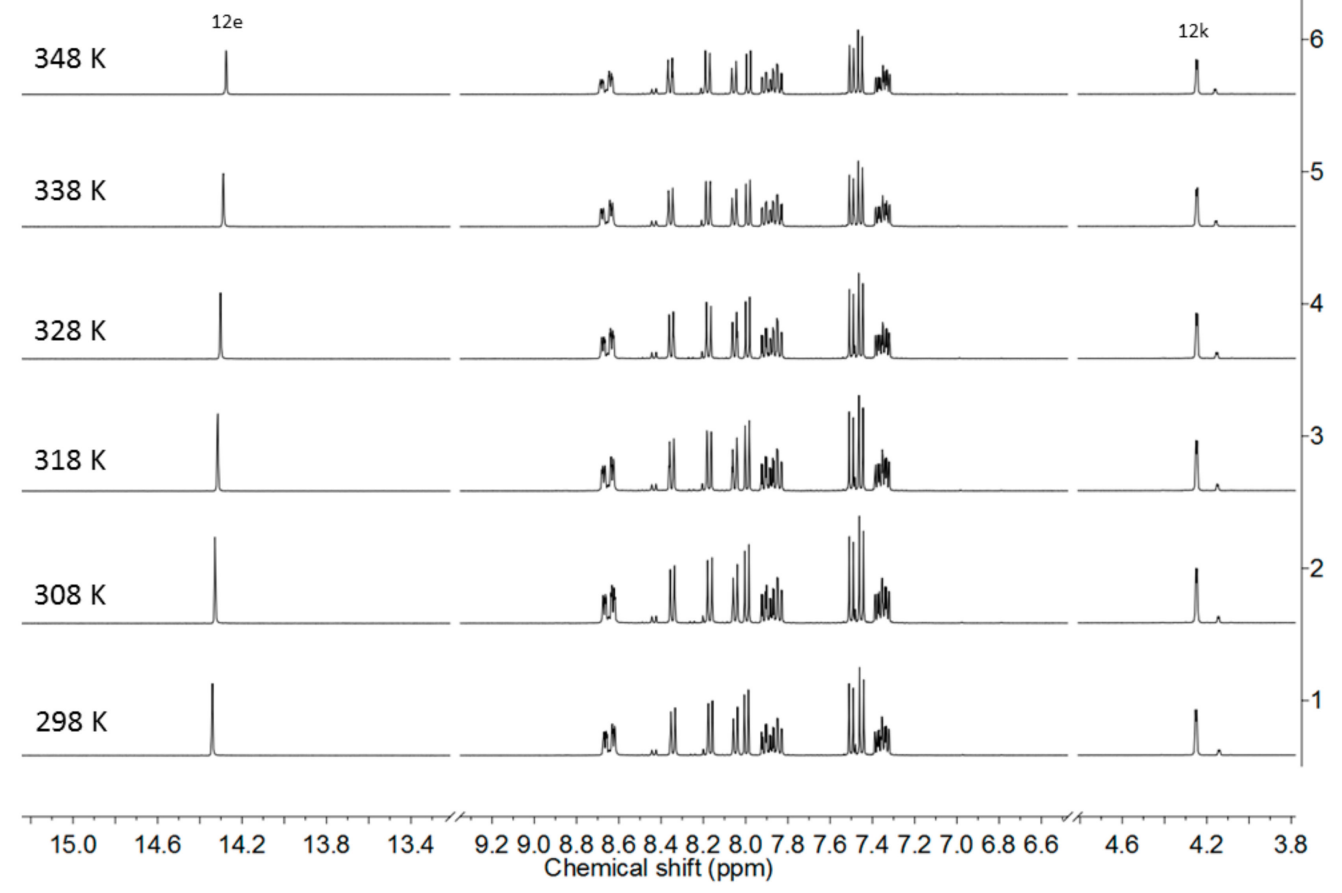
High shift region of the variable temperature (VT) ^1^H-NMR spectra of Compound **3** in CD_3_CN.

Due to the fact that the current keto-enol tautomerism was accompanied by substantial structural rearrangement, it was slow on the NMR timescale. As a result, two sets of signals corresponding to the tautomers were obtained in various deuterated solvents, such as decalin-*d18,* CD_2_Cl_2_, CDCl_3_, and CD_3_CN (Figure 2 and Figures S12–S14). In CD_3_CN, the characteristic signal H12e belonging to the OH group of the enol form **3e** appeared at 14.30–14.50 ppm, whereas the signal characteristic of the keto form (H12k) appeared at around 4.30 ppm (Figure 2). The ^1^H-NMR shifts of the two signals (H12k and H12e) were in good agreement with the calculated ones. Considering the H-bond with the N atom of the pyridine unit, values of 4.66 ppm for H12k and 14.16 ppm for H12e were obtained. The slight deviation observed for H12k (Δ = 0.36 ppm) could indicate a rotation around the single bond, which would shift the proton upfield, as observed experimentally. For instance, according to the calculations, the **3k** tautomer existed as two stable isomers with near energies (rotation around the single OC-CH12k-bond) where the ^1^H shift of H12k varied from 4.10 to 4.66 ppm. In the VT ^1^H-NMR spectra, the shift of the H12e proton changed. As the temperature increased from 298 to 348K, the signal shift decreased by 0.07 ppm, indicating the weakening of the O-H-bond with temperature (Figure 2). The keto form **3k** was also proven experimentally by the presence of a signal at 210 ppm (C=O) in the ^13^C-NMR spectrum (Figure S9).

The existence of two distinct sets of ^1^H-NMR signals for **3e** and **3k** allowed a quantitative estimation of the tautomeric ratio, allowing evaluation of the equilibrium constant K_T_ = [enol]/[keto] and of the Gibbs free energy (ΔG°), enthalpy (ΔH°), and entropy (ΔS°) values. Depending on solvent polarity, different ΔG° values were obtained (Table 1). The relative stability of the two forms seemed to be influenced by solvent polarity, as well as water content (Figure S1). The less polar enol **3e** was favored in less polar solvents, while the presence of water favored the keto form **3k** through intermolecular H-bond formation between the free carbonyl group of **3k** and water. By plotting ΔG° = ΔH° − T ΔS° (Figure S2), the ΔH° and ΔS° could also be calculated in CD_3_CN. The obtained values were −0.83 kcal/mol for ΔH° and −0.003 kcal/mol·K for ΔS°. This clearly indicated that the equilibrium **3k**–**3e** was mainly enthalpy driven. Thus, a temperature decrease favored the enol form **3e**.

The data in Table 1 show a reasonable agreement between theory and experiment. The tautomeric equilibrium was correctly predicted by theoretical calculations. Moreover, the decrease in the solvent polarity led to destabilization of the more polar **3k**, as expected. The only deviation observed was the relative order of experimentally determined values of ΔG° in CD_3_CN and chlorinated solvents. It seemed that the more polar keto tautomer was better stabilized by the less polar (compared to CD_3_CN) CD_2_Cl_2_ and CDCl_3_. The chlorinated solvents were in fact strong proton donor solvents; thus, the keto tautomer **3k** could be more favored through intermolecular H-bonding involving the free carbonyl group, as reported in other tautomeric systems [23,24]. 

**Scheme 2:**
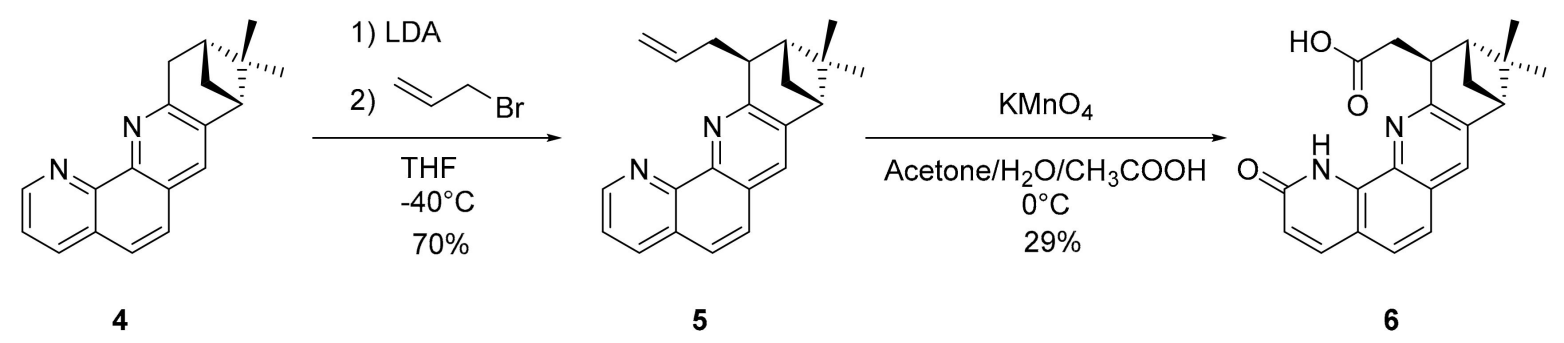
Synthetic pathway for **6**.

The second studied compound, pinene-phenanthroline-methylene-carboxylate **6**, was obtained in two steps from Compound **4** [7]. Deprotonation of **4** followed by an alkylation with allyl bromide gave the intermediate **5** (Scheme 2 and Figures S15 and S16). This intermediate **5** was subsequently oxidized with KMnO_4_. To our surprise, besides the oxidation of the allyl unit to the carboxylic acid, the phenanthroline moiety was also oxidized. This was a rather unusual oxidation, as most of the oxidations of the 1,10-phenantroline affected the CH groups from the central condensed cycle [25]. 

The theoretical calculations predicted that the other tautomeric forms of the **6** were substantially less stable compared with the tautomeric form observed experimentally (Scheme 2 and Table S1). This stabilization, being typical for 2- and 4-hydroxy-1,10- phenanthroline, was a result of the formed intramolecular hydrogen N…H-N-bonding [26,27].

**Figure 3:**
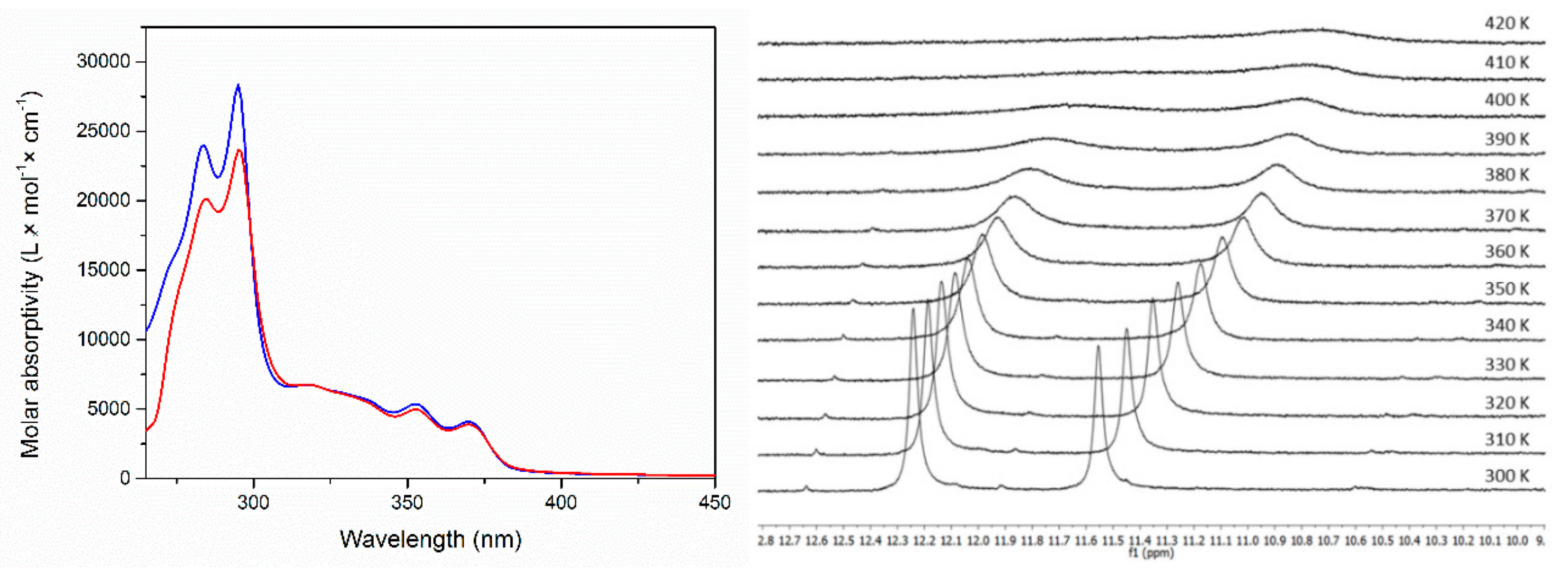
Left: Absorption spectra of **6** in DMSO as a function of the concentration while keeping the product between the concentration value and the path length constant (5.0 × 10^−5^ M). The concentrations are as follows: blue solid line: 5.0 × 10^−4^ M (most concentrated); red solid line: 5.0 × 10^−6^ M (most diluted). Right: High shift region from variable temperature ^1^H-NMR spectra of Compound **6**, in DMSO-d6.

During the oxidation process, the aromaticity of one condensed cycle was broken in Compound **6**, as depicted in Scheme 2. Due to the presence of lactam and carboxylic acid moieties, the compound aggregated, as observed through UV-Vis spectroscopy (Figure 3 and Figure S4) and ^1^H-NMR spectroscopy (S17, S18). In the UV-Vis spectra in DMSO, the band at ~290 nm lowered with dilution, while the long wavelength peak showed little change. The spectra in can were featured in the same way, which indicated that there was no substantial solvent effect. According to the NMR data, the dimerization took place through intermolecular H-bonding between the amide group and the carboxylic unit from a neighboring molecule forming a cyclic structure. At 300 K, in the ^1^H-NMR spectrum in DMSO-*d6*, the proton signal from the COOH appeared at 12.24 ppm, while the one of the NH group appeared at 11.55 ppm (Figure 3 and Figure S19). When the temperature was increased, the signal corresponding to the COOH unit disappeared, while the NH signal broadened and shifted upfield to 10.73 ppm. These observations showed that a temperature increase broke the H-bonds, which led to the formation of the monomeric form. 

**Figure 4:**
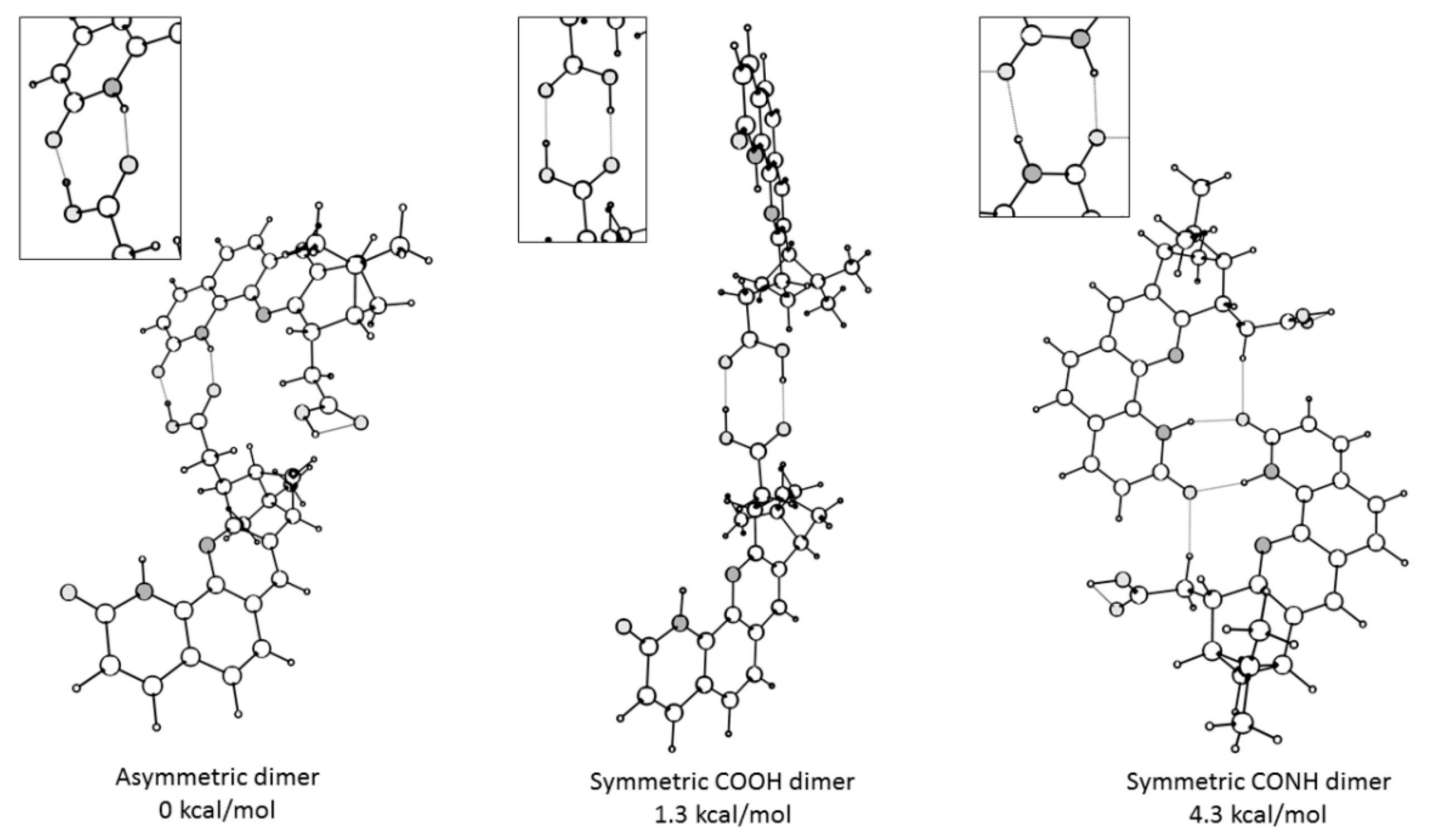
Possible dimers of **6** in DMSO with their relative energies estimated without counterpoise corrections. The differences in the gas phase with counterpose correction are as follows: 0, 1.5 and 2.5 kcal/mol.

**Figure 5:**
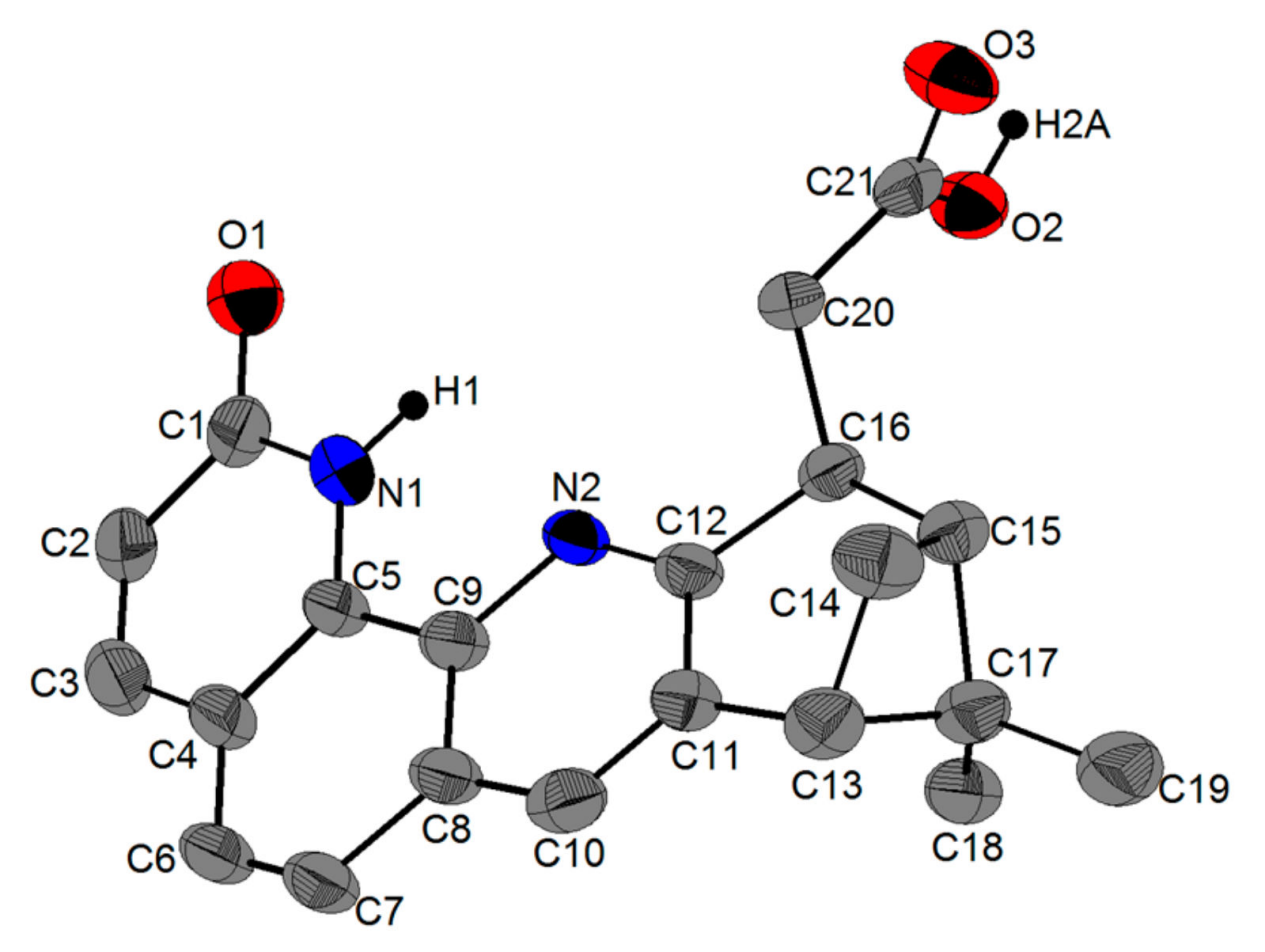
View of the molecular structure of **6** as determined by single crystal X-ray diffraction, at 30% probability. The H atoms connected to carbon atoms have been removed for clarity.

**Figure 6:**
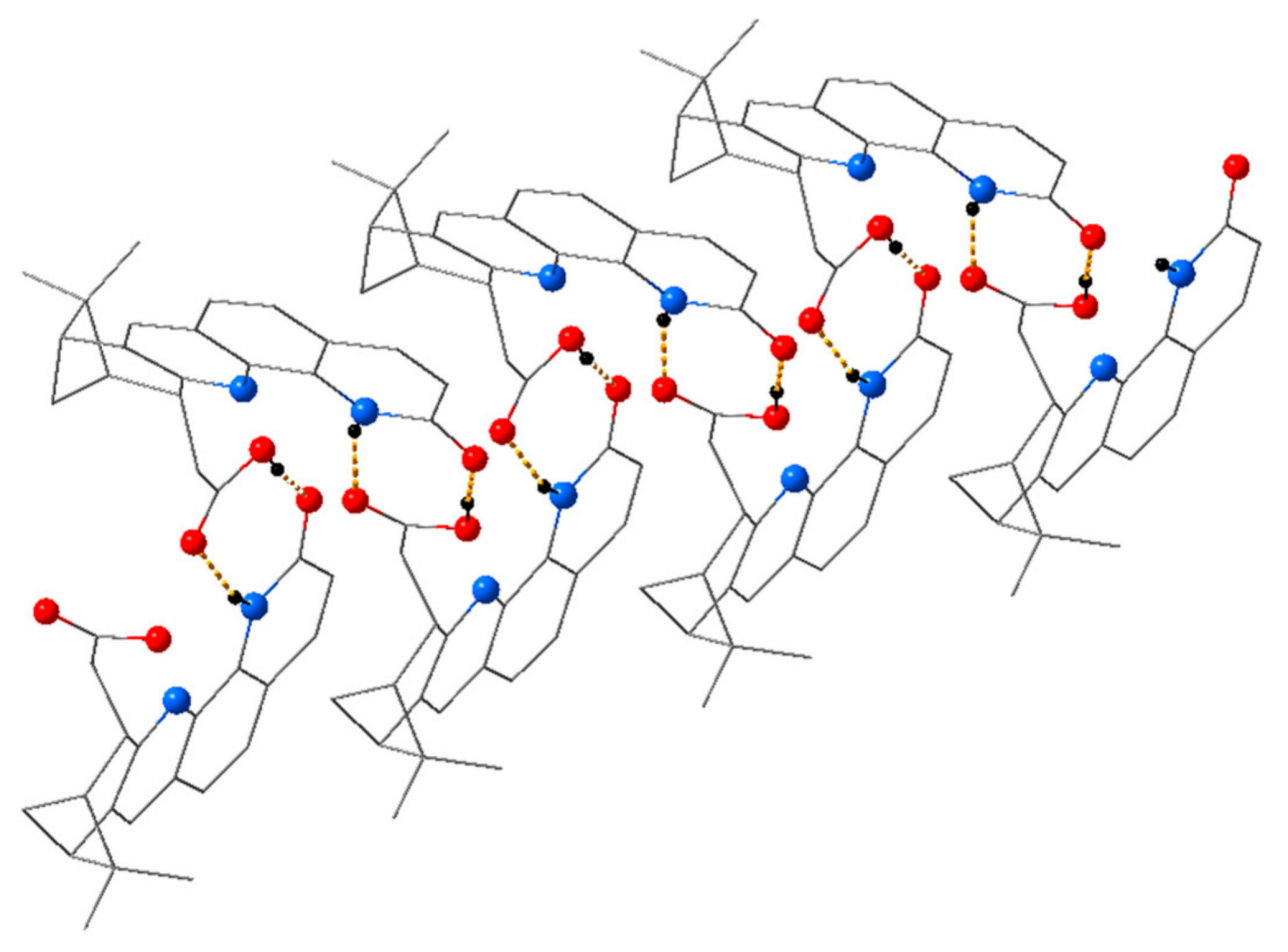
Hydrogen bonding pattern in the solid state structure of **6**.

This was in a good agreement with the theoretical calculations showing that the most stable dimer of Compound **6** was the asymmetric one, formed through interaction between COOH and CONH groups (Figure 4). Moreover, it was also in an excellent agreement with the X-ray structure (Figure 5) where a helical-like arrangement of the molecules connected via intermolecular H-bonds between the carboxylic and amido moieties was observed (Figure 6). This type of aggregate explained the observed spectral changes. According to the predicted spectra of the monomer and dimer (Table S2), there was little difference between the long wavelength bands both as position and intensity. Therefore, the dilution did not lead to substantial changes in the 310–400 nm region. However, the intensity of the band around 290 nm was substantially large in the dimer, which led to the rise of this band upon the concentration rise. The predicted ^1^H-NMR shifts for the protons involved in the H-bonding in the dimer were 14.0 (NH) and 15.5 ppm (COOH), while those of a single molecule should be 6.71 (COOH) and 10.25 ppm (NH). The difference between the calculated shifts assuming that solely dimers were formed and those experimentally measured by ^1^H-NMR indicated that the actual aggregation in solution was more complicated and probably led to the formation of higher order aggregates. This corroborates the unusually high value of the specific rotation ${\left[\alpha \right]}_{D}^{20}$ = +359 deg∙cm^3^∙dm^−1^∙g^−1^ measured for the molecule **6**. Indeed, such high specific rotation can be due to the Horeau effect, which arises when chiral molecules aggregate in solution, forming supramolecular species with higher rotatory power than the monomers [28].

### Single Crystal X-Ray Diffraction Studies

Compound **6** crystallized in the space group *P*2_1_2_1_2_1_ (N° 19) with one molecule in the asymmetric unit. The solid state structure of **6** was in good agreement with the solution data as well. Only the lactam tautomer drawn in Scheme 2 was present in the solid state, as shown by the C–N and C—O bond lengths. In the lactam ring, the C1—C2- and C3—C4-bonds had lengths of 1.426(10) Å and 1.430(11) Å, respectively, which were typical for single C-C bonds. The C2—C3- and C4—C5-bonds showed a double bond character, with lengths of 1.333(11) Å and 1.386(9) Å, respectively. The C1—O1-bond also showed a double bond character with a bond length of 1.274(9) Å. 

Each molecule was interacting with a neighboring molecule through H-bonding between the lactam moiety and the carboxylic unit, forming a $R_{2}^{2}\left(8\right)$ motif. The ring constraint forced the lactam unit into the *cis* configuration necessary for such a type of H-bonding pattern. The O1∙∙∙H2A—O2 distance was 1.782(6) Å, and the O1—H2A—O2 angle was 171.2°, while the N1—H1∙∙∙O3 distance measured 2.045(6) Å, with an angle N1—H1—O3 of 160.2°. These distances and angles indicated a moderately strong H-bond [29]. The intermolecular H-bonds led to a helical, screw-like arrangement of the molecules in the crystal, as shown in Figure 6.

## 3. Materials and Methods

### 3.1. General

If inert conditions were needed, the reactions were performed under a nitrogen atmosphere using oven dried glassware (120 °C). Analytical thin layer chromatography (TLC) was performed on SiO_2_ plates GF254 (0.25 mm layer thickness). Flash chromatography purifications were performed on a CombiFlash EZ Prep from Teledyne. ^1^H-NMR and ^13^C-NMR spectra were recorded on Bruker Avance DPX 300 and Bruker Avance 400 MHz spectrometers, using TMS or the residual solvent proton as the internal standard. Coupling constants are reported in Hz. (+)-ESI-MS spectra were recorded on the Bruker FT-MS 4.7T Bio Apex II instrument.

#### 3.1.1. Single Crystal X-Ray Diffraction of **6**

Colorless needle shaped single crystals of **6** were recrystallized from acetone by slow evaporation. A suitable crystal of size 0.32 × 0.14 × 0.02 mm^3^ was selected and mounted on a mylar loop in oil on a Stoe IPDS2 diffractometer. The crystal was kept at a steady *T* = 250(2) K during data collection. The structure was solved with the ShelXT [30] structure solution program using the Intrinsic Phasing solution method and by using Olex2 [31] as the graphical interface. The model was refined with Version 2017/1 of ShelXL [32] using least squares minimization. CCDC 1,958,303 contains the supplementary crystallographic data for **6**. These data can be obtained free of charge via http://www.ccdc.cam.ac.uk/conts/retrieving.html, or from the Cambridge Crystallographic Data Centre, 12 Union Road, Cambridge CB2 1EZ, U.K.; fax: (+44) 1223-336-033; or e-mail: deposit@ccdc.cam.ac.uk.

The starting materials were purchased from Sigma-Aldrich, Fluka, or Acros Organics and used without further purification, unless stated otherwise. **1 [10]** and **4** [7] were synthesized as previously reported in the literature, starting from 81% ee pinocarvone.

#### 3.1.2. Synthesis of **2**

Under inert atmosphere, anhydrous THF (15 mL) and diisopropylamine (0.38 mL, 2.65 mmol, 1.325 eq) were added into a two neck flask and subsequently cooled to 0 °C. Over this, nBuLi (1.15 mL, 2.5 mmol, 1.25 eq, 2.2 M solution in hexane) was added dropwise. The solution was stirred at this temperature for 10 minutes and afterwards cooled to −40 °C. A solution of (−)-5,6-pinene-bipyridine (500 mg, 2 mmol, 1 eq) in dry THF (8 mL) was added dropwise. The dark blue solution was stirred at −40 °C for 2 h. CH_3_CHO (0.17 mL, 3.0 mmol, 1.5 eq) was then added, and the solution was allowed to warm to room temperature and afterwards stirred for an additional 30 min. The obtained light brown mixture was quenched with HCl (0.3 mL, 1M). The solvent was evaporated under reduced pressure, and the residue was dissolved in CH_2_Cl_2_ (30 mL). The mixture was washed with water (3 × 10 mL), and the collected organic phases were dried over anhydrous MgSO_4_. The solvent was removed under reduced pressure, and the obtained oil was further purified by column chromatography on silica gel using n-hexanes:Et_2_O:Et_3_N (4:1.5:0.25) as the eluent. Compound **2** (Rf = 0.4) was obtained as a light yellow oil (0.25 g, yield 45%). ^1^H-NMR (300 MHz, CDCl_3_) δ = 8.66 (m,1H, H1), 8.20 (m, 2H, H4, H5), 7.77 (m, 1H, H3), 7.43 (d, *J* = 7.8 Hz, 1H, H6), 7.31–7.27 (m, 1H, H2), 7.22 (s, 1H, OH), 4.36 (m, 1H, H12B), 4.09 (m, 1H, H12A), 3.11 (dd, *J* = 9.5, 2.4 Hz, 1H, H9B), 2.94 (dd, *J* = 9.3, 2.5 Hz, 1H, H9A), 2.88–2.73 (m, 3H, H14A, H14B, H7B), 2.60 (dt, *J* = 10.0, 5.7 Hz, 1H, H8Aa), 2.54 (td, *J* = 6.2, 2.4 Hz 1H, H8Ba), 2.36 (td, *J* = 6.0, 2.5 Hz, 1H, H7A), 1.45 (s, 3H, H10A), 1.44 (s, 3H, H10B), 1.42 (d, *J* = 8.9 Hz, 1H, H8Bb), 1.35 (d, *J* = 9.9 Hz, 1H, H8Ab), 1.31 (d, *J* = 6.1 Hz, 3H, H13B), 1.30 (d, *J* = 6.1 Hz, 3H, H13A), 0.70 (s, 3H, H11B), 0.69 (s, 3H, H11A). ^13^C-NMR (75 MHz, CDCl_3_) δ = 159.13, 158.82, 155.77, 155.71, 152.48, 149.33, 142.66, 142.05, 137.16, 134.76, 134.64, 123.61, 123.60, 120.66, 119.03, 118.99, 69.59, 69.23, 55.05, 50.15, 47.06, 46.91, 43.38, 42.44, 41.46, 39.55, 33.76, 28.92, 26.91, 26.34, 24.13, 21.29, 21.10, 21.09. ${\left[\alpha \right]}_{D}^{20}$ = −5.3 deg∙cm^3^∙dm^−1^∙g^−1^ (c 0.184 g/L, CH_2_Cl_2_). ESI-MS (+): 295.1 [M + H]^+^ and 317.0 [M + Na]^+^. HRMS (ESI) calcd. for C_19_H_23_H_2_O ^+^ [M + H]^+^ 295.1805, found 295.1793.

#### 3.1.3. Synthesis of **3**

Under inert atmosphere, (COCl)_2_ (0.75 mL, 8.56 mmol, 1.4 eq) was dissolved in CH_2_Cl_2_ (45 mL). The solution was cooled to −78 °C. DMSO (1.30 mL, 17.12 mmol, 2.8 eq) was added dropwise. The solution was stirred at this temperature for 15 min. A solution of **1** (1.80g, 6.114 mmol, 1eq) in anhydrous CH_2_Cl_2_ (45 mL) was added dropwise. The reaction mixture was allowed to reach room temperature and stirred for 2.5 h. Then, the solvent was evaporated under reduced pressure. EtOAc (100 mL) was added, followed by a saturated solution of Na_2_CO_3_ (75 mL). The organic phase was washed with brine and dried over Na_2_SO_4_. The solvent was evaporated under reduced pressure, and the residue was purified by column chromatography on silica gel, using n-hexane:EtOAc (4:0.5) as the eluent. Compound **3** (1.08 g, 60% yield, Rf = 0.2) was obtained as an orange oil. ^1^H-NMR (400 MHz, CD_3_CN) δ = 14.34 (m, 1H, H12e ), 8.67 (ddd, *J* = 4.8, 1.8, 1.0 Hz, 1H, H1k), 8.63 (ddd, *J* = 4.8, 1.8, 0.9 Hz, 1H, H1e), 8.34 (dt, *J* = 8.0, 1.1 Hz, 1H, H4k), 8.17 (dd, *J* = 7.8, 0.7 Hz, 1H, H5k), 8.05 (dt, *J* = 8.0, 1.1 Hz, 1H, H4e), 8.00 (d, *J* = 7.7 Hz, 1H, H5e), 7.90 (td, *J* = 7.8, 1.8 Hz, 1H, H3e), 7.85 (td, *J* = 7.9, 1.8 Hz, 1H, H3k), 7.50 (d, *J* = 7.7 Hz, 1H, H6e), 7.45 (d, *J* = 7.8 Hz, 1H, H6k), 7.39–7.35 (m, 1H, H2e), 7.35–7.31 (m, 1H, H2k), 4.25 (d, *J* = 2.4 Hz, 1H, H12k), 3.01 (t, *J* = 6.0 Hz, 1H, H7e), 2.95 (t, *J* = 5.8 Hz, 1H, H9e), 2.86 (t, *J* = 5.7 Hz, 1H, H7k), 2.83–2.76 (m, 1H, H8e-b), 2.65 (dt, *J* = 10.0, 5.4 Hz, 1H, H8k-b), 2.58–2.54 (m, 1H, H9k), 2.43 (s, 3H, H13k), 2.00 (d, *J* = 0.8 Hz, 3H, H13e), 1.50 (d, *J* = 10.1 Hz, 1H, H8k-a), 1.47 (s, 3H, H10k), 1.46 (s, 3H, H10e), 1.31 (d, *J* = 9.4 Hz, 1H, H8e-a), 0.65 (s, 3H, H11k), 0.62 (s, 3H, H11e). ^13^C-NMR (126 MHz, CD_3_CN) δ = 209.48, 158.15, 157.04, 156.72, 155.86, 155.18, 154.34, 150.50, 150.15, 143.72, 140.51, 138.34, 137.93, 135.25, 134.84, 124.65, 124.48, 121.19, 120.71, 119.18, 118.29, 117.35, 108.73, 58.53, 48.43, 46.74, 45.38, 44.15, 42.85, 40.93, 32.94, 31.39, 29.83, 26.18, 26.11, 22.56, 21.18, 18.14. ${\left[\alpha \right]}_{D}^{20}$= −60 deg∙cm^3^∙dm^−1^∙g^−1^ (c 0.100 g/L, CH_2_Cl_2_). HRMS (ESI) calcd. for C_19_H_21_N_2_O^+^ [M+H]^+^ 293.1648, found 293.1653.

#### 3.1.4. Synthesis of **5**

Using the standard Schlenk technique, diisopropylamine (0.28 mL, 2 mmol, 1.1 eq) was added to anhydrous THF (30 mL) in a 100 mL Schlenk flask. The solution was cooled to −40 °C, and a solution of nBuLi (1.6 M, 1.2 mL, 1.8 mmol, 1 eq) was added. Compound **4** (0.5 g, 1.8 mmol, 1 eq) dissolved in anhydrous THF (30 mL) was added dropwise over 15 min. A very deep red color appeared instantly. This solution was stirred for 1 h at −40 °C. Allyl bromide (0.3 mL, 3.6 mmol, 2 equiv) was then added, and the cooling bath was removed. Stirring was continued overnight. Methanol (2 mL) was then added, and the reaction mixture was evaporated and purified on silica gel using tBuOMe:Et_3_N (95:5) as the eluent. Compound **5** (0.42 g, 70% yield, Rf = 0.6) was obtained as a beige solid. ^1^H-NMR: (300 MHz, CDCl_3_) δ = 9.11 (dd, *J* = 4.4, 1.7 Hz, 1H, H1), 8.13 (dd, *J* = 8.1, 1.7 Hz, 1H, H3), 7.63 (m, 3H, H6, H7, H10), 7.49 (dd, *J* = 8.1, 4.4 Hz, 1H, H2), 5.92 (m, 1H, H21), 5.02 (m, 2H, H22), 3.50 (m, 2H, H20), 2.94 (t, *J* = 5.7 Hz, 1H, H13), 2.55 (m, 1H, H14a), 2.36 (td, *J* = 6.1, 2.4 Hz, 1H, H15), 2.24 (m, 1H, H16), 1.4 (s, 3H, H19), 1.36 (dd, *J* = 9.8 Hz, 1H, H14b), 0.25 (s, 3H, H18). ^13^C-NMR (75 MHz, CDCl_3_) δ = 161.53, 150.30, 146.17, 144.43, 142.22, 137.75, 135.95, 131.17, 128.19, 127.15, 126.43, 125.65, 122.10, 116.11, 47.40, 44.31, 42.64, 41.01, 37.51, 26.37, 21.08. ${\left[\alpha \right]}_{D}^{20}$ = −53 deg∙cm^3^∙dm^−1^∙g^−1^ (c 7.900 g/L, CH_2_Cl_2_). HRMS (ESI) calcd. for C_22_H_22_N_2_^+^ [M + H]^+^ 315.1783, found 315.1849. 

#### 3.1.5. Synthesis of **6**

A mixture of Compound **5** (0.3 g, 1 mmol, 1 eq), acetone (6 mL), acetic acid (3 mL), and water (2 mL) was stirred at 0 °C. KMnO_4_ (0.8 g, 5 mmol, 5 eq) was then slowly added over 3 h. H_2_O_2_ (5 mL, 30%) was added dropwise at 0 °C giving rise to a strong foam. The mixture was then extracted with CH_2_Cl_2_ (3 × 50 mL), and the combined organic phases were washed with water (3 × 50 mL). Solvents were evaporated, and the residue was purified using a gradient of eluent n-heptane:EtOAC (6:4) and then EtOAc (100%). Compound **6** (0.10g, 29% yield, Rf = 0.5) was obtained as a light yellow solid. ^1^H-NMR: (300 MHz, CDCl_3_) δ = 13.13 (s, 1H, H21), 7.98 (d, *J* = 9.4 Hz, 1H, H3), 7.68 (s, 1H, H10), 7.54 (dd, *J* = 21.8, 8.6 Hz, 2H, H6, H7), 6.86 (d, *J* = 9.3 Hz, 1H, H2), 4.12 (q, *J* = 7.0 Hz, H15), 3.97 (m, 1H, H16), 3.03 (t, *J* = 5.6 Hz, 1H, H13), 2.80 (m, 1H, H14a), 2.64 (m, 2H, H20), 1.48 (d, *J* = 9.1 Hz, 1H, H14b), 1.46 (s, 3H, H19), 0.72 (s, 3H, H18). ^13^C-NMR (75 MHz, CDCl_3_) δ= 178.09, 164.39, 160.49, 143.11, 141.46, 135.73, 135.36, 130.73, 127.29, 124.50, 121.51, 121.47, 117.56, 47.65, 43.36, 42.77, 41.36, 38.02, 28.96, 26.34, 21.27. IR: (ATR, cm^−1^) 3161w(br), 2927m, 2542w(br), 1614s, 840s(sh) ${\left[\alpha \right]}_{D}^{20}$ = +359 deg∙cm^3^∙dm^−1^∙g^−1^ (c 4.800 g/L, CH_2_Cl_2_). HRMS (ESI) calcd. for C_21_H_20_N_2_O_3_^+^ [M + H]^+^ 349.1547, found 349.1536. Crystal data: C_21_H_20_N_2_O_3_, *M_r_* = 348.39, orthorhombic, *P*2_1_2_1_2_1_ (No. 19), a = 6.2818(8) Å, b = 14.5974(17) Å, c = 19.054(3) Å, *a* = *b* = *g* = 90°, *V* = 1747.2(4) Å^3^, *T* = 250(2) K, *Z* = 4, *Z′* = 1, *µ*(MoK*_a_*) = 0.089, 25,010 reflections measured, 3517 unique (*R_int_* = 0.2809), which were used in all calculations. The final *wR*_2_ was 0.0936 (all data), and *R*_1_ was 0.0462 (I > 2(I)).

#### 3.1.6. Quantum-Chemical Calculations

Quantum-chemical calculations were performed by using the Gaussian 09 program suite [33]. The M06-2X functional [34,35] was used with the TZVP basis set [36]. This fitted hybrid meta-GGA functional with 54% HF exchange was especially developed to describe main group thermochemistry and the non-covalent interactions, showing very good results in the prediction of the position of the tautomeric equilibrium in azo naphthols possessing intramolecular H-bonds [37] and in the description of the proton transfer reactions in naphthols [38,39]. All structures were optimized without restrictions, using tight optimization criteria and an ultrafine grid in the computation of two electron integrals and their derivatives, and the true minima were verified by performing frequency calculations in the corresponding environment. Solvent effects were described by using the polarizable continuum model (the integral equation formalism variant, IEFPCM, as implemented in Gaussian 09) [40]. 

The optimized at the M06-2X/TZVP level structures of tautomers and/or dimers were used without further optimization for absorption spectra and NMR chemical shieldings’ prediction in the same solvent by changing the basis set to B3LYP/6-311+G(2d,p). The absorption spectra of the compounds were predicted using the TD-DFT formalism [41,42,43]. The NMR chemical shieldings were calculated using the GIAO approximation [44] at the B3LYP/6-311+G(2d,p) level of theory. This level of theory was recommended in the pioneering work by Cheeseman et al. [45] focused on the comparison of different models for calculating nuclear magnetic resonance shielding tensors and showed very good results in predicting the NMR spectra of azo-hydrazone tautomerism [46]. The calculated absolute shieldings were transformed to chemical shifts using the reference compound tetramethylsilane, Si(CH_3_)_4_, for hydrogen: δ = δ_calc_(ref) − δ_calc_. Both δ_calc_(ref) and δ_calc_ were evaluated at the same computational level.

In the case of dimers, counterpoise corrections were computed in the gas phase using the methodology integrated [47] in the Gaussian 09 program suite.

## 4. Conclusions

In conclusion, the tautomerism and self-association of two novel ligands derived from pinene were characterized spectroscopically and corroborated with the theoretical calculations obtained by DFT. The keto-enol tautomerism of Compound **3** was studied by means of DFT calculations, ^1^H-NMR, and UV-Vis spectroscopy. The existence of two distinct sets of signals for each of the tautomers allowed a quantitative estimation of the tautomeric ratio, giving access to the K_T_ = [enol]/[keto], ΔG°, ΔH°, and ΔS° values. Depending on the solvent, the ΔG° values were in the range of −0.46 to 0.28 kcal/mol, in good agreement with the calculated values. 

Although Compound **6** was potentially tautomeric, it existed as a single lactam tautomer due to the high energy difference between the two potential tautomers. The compound aggregated through H-bonding between the lactam moiety and the carboxylic acid, as shown by ^1^H-NMR, single crystal X-ray diffraction and DFT calculations. 

## Figures and Tables

**Table 1:** Relative energies ΔE (from the theoretical calculations) and free energies ΔG° (from ^1^H-NMR spectra) of the tautomer **3k** relative to **3e** in various solvents. A positive value indicates that the keto form is more stable.

Solvent	Theoretical ΔE (kcal/mol)	Experimental ΔG° (kcal/mol)
CD_3_CN	0.84	0.15
CD_2_Cl_2_	0.37	0.28
CDCl_3_	−0.06	0.19
decalin-*d18*	−0.82	−0.46

## Supplementary Materials

The following are available online: Figure S1: Influence of the water content on the keto-enol equilibrium **3k ⇌ 3e** (62 mM in CD_3_CN); Figure S2. Dependence of the free enthalpy (ΔG°) as a function of temperature for Compound **3** ((62 mM in CD_3_CN), from VT ^1^H-NMR experiments; Figure S3. Absorption spectra of **3** in organic solvents; Figure S4. Absorption spectra of **6** in CH_3_CN while keeping the product of the concentration value and the path length constant; Figures S5–S19: NMR spectra; Figures S19–S26: MS spectra; Table S1. The most stable isomers of the existing tautomers of **6** in chloroform; Table S2. Predicted absorption spectra of the monomer and dimer of **6** in DMSO; Table S2. Predicted absorption spectra of the monomer and dimer of **6** in DMSO; Table S3. Crystallographic data for **6**.
