# Supplementary material for: Tautomerism and Self-Association in the Solution of New Pinene-Bipyridine and Pinene-Phenanthroline Derivatives
Source: Molecules. 2020 Jan 11;25(2):298. doi: 10.3390/molecules25020298 (PMC7024227; doi:10.3390/molecules25020298)
Supplement: Supplementary file 1 [file molecules-25-00298-s001.pdf]

Supporting Information

Tautomerism and Self-Association in the Solution of New Pinene-Bipyridine and Pinene-Phenanthroline Derivatives

Atena B. Solea [1,2], Ivan Cornu [1], Vera Deneva [3], Aurelien Crochet [1], Katharina M. Fromm [2], Liudmil Antonov [3], Christophe Allemann [1] and Olimpia Mamula [1,*]

1 Haute Ecole d'Ingénierie et d'Architecture Fribourg, HES-SO University of Applied Sciences of Western Switzerland, Pérolles 80, CH-1705 Fribourg, Switzerland; Atena-Bianca.Solea@hefr.ch (A.B.S.); ivan.cornu@master.hes-so.ch (I.C.); aurelien.crochet@unifr.ch (A.C.); Christophe.Allemann@hefr.ch (C.A.)

2 Department of Chemistry, University of Fribourg, Chemin du Musée 9, CH-1700 Fribourg, Switzerland; katharina.fromm@unifr.ch (K.M.F.)

3 Bulgarian Academy of Sciences, Institute of Organic Chemistry with Centre of Phytochemistry, Acad. G. Bonchev street, bl. 9, 1113 Sofia, Bulgaria; vdeneva@orgchm.bas.bg (V.D.); Lantonov@orgchm.bas.bg (L.A.)

* Correspondence: olimpia.mamulasteiner@hefr.ch

Contents

^{1}H-NMR, ^{13}C-NMR Spectra 6

Mass spectra 14

Crystallographic data for **6** .... **Error! Bookmark not defined.**

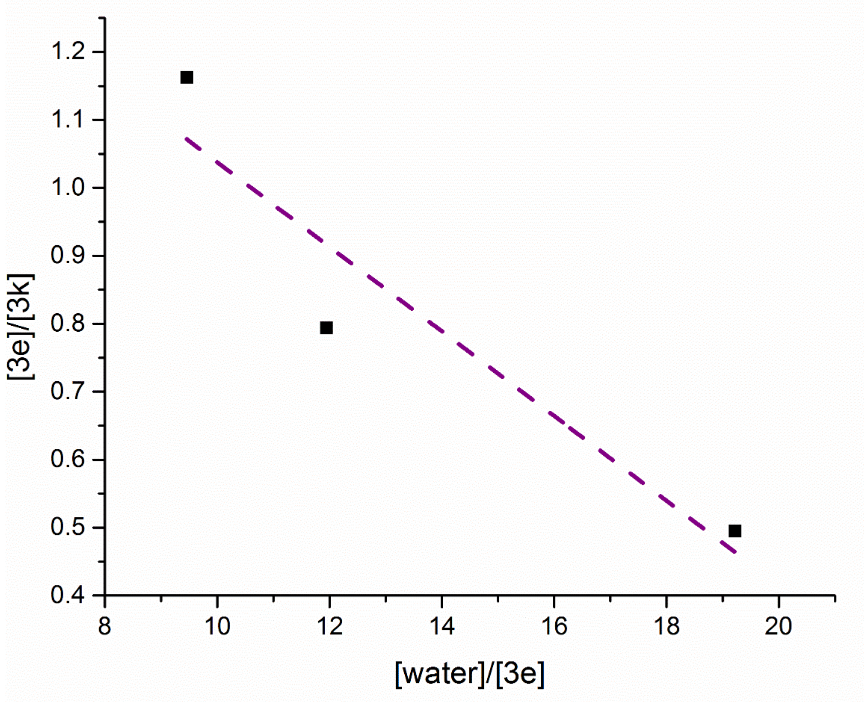

Figure S1. Influence of the water content on the keto-enol equilibrium **3k** ⇌ **3e** (62 mM in CD_3CN)

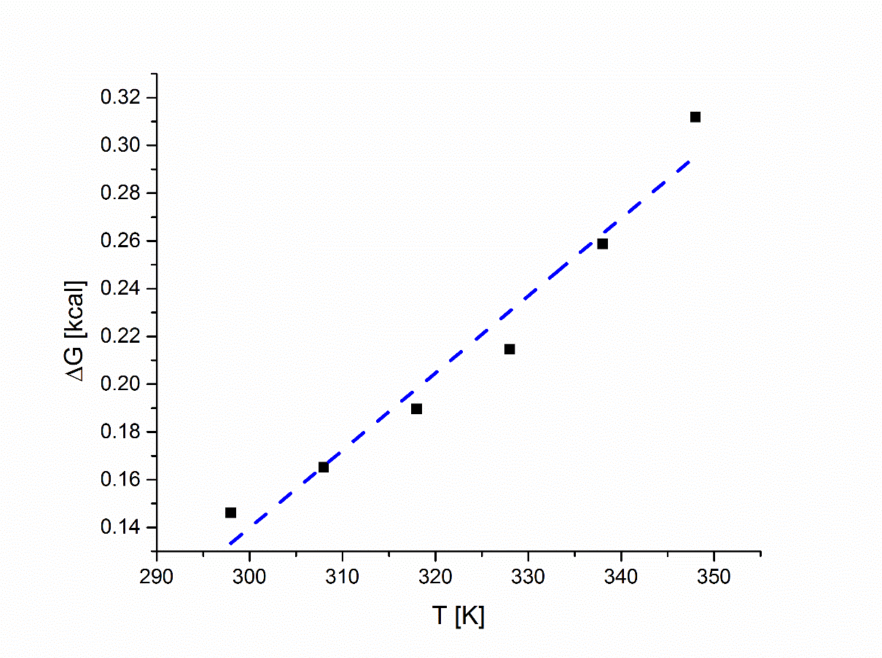

Figure S2. Dependence of the free enthalpy (ΔG°) as a function of temperature for compound **3** (62 mM in CD_3CN), from VT ^{1}H-NMR experiments

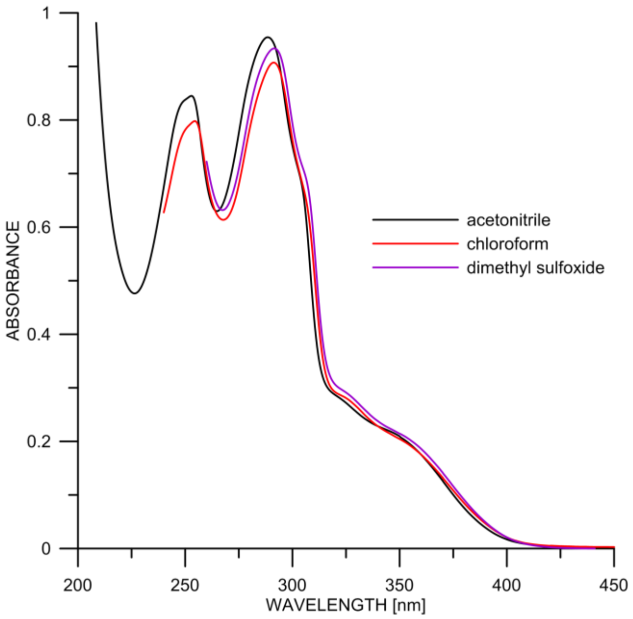

Figure S3. Absorption spectra of **3** in organic solvents.

Table S1. Most stable isomers of the existing tautomers of **6** in chloroform.

Tautomer	ΔE, kcal/mol	μ, D
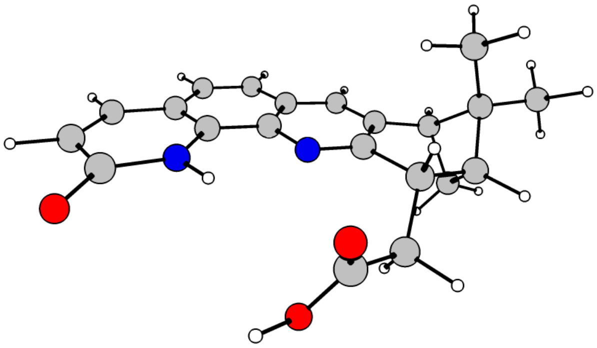	0	9.6
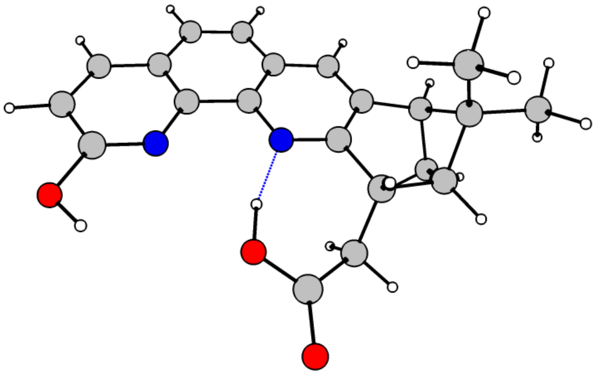	3.0	10.0

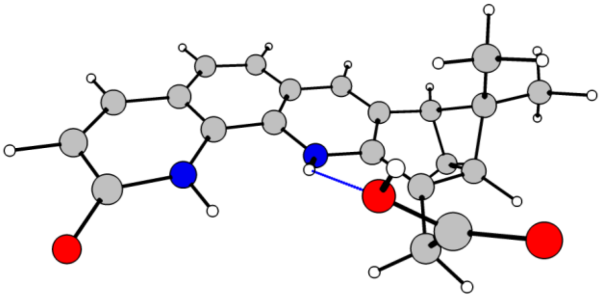	21.4	6.1
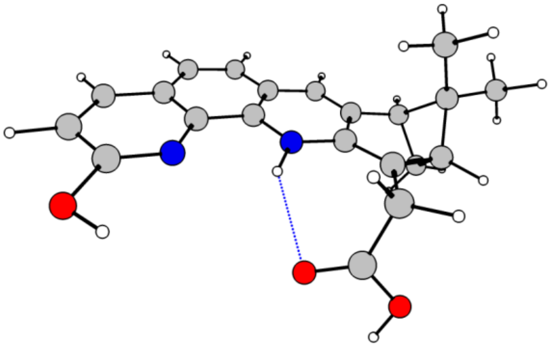	21.1	2.1
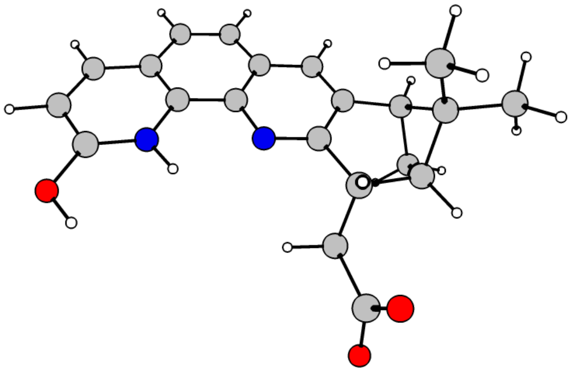	35.4	29.2
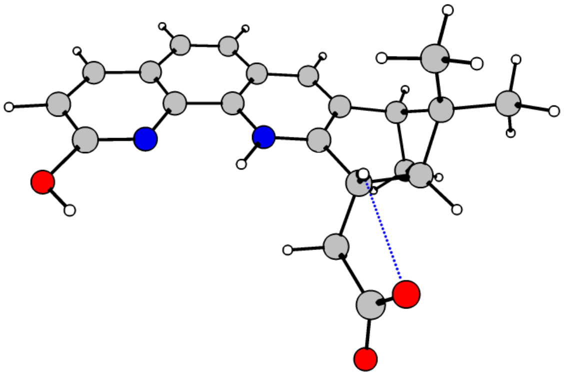	25.3	24.8

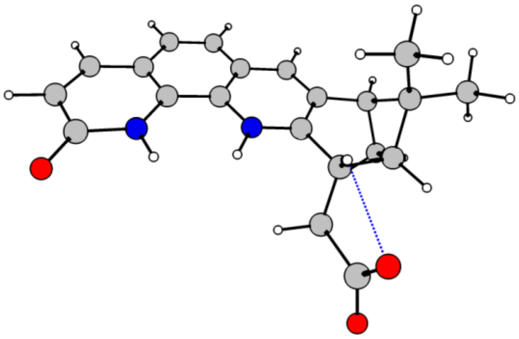	32.1	25.8

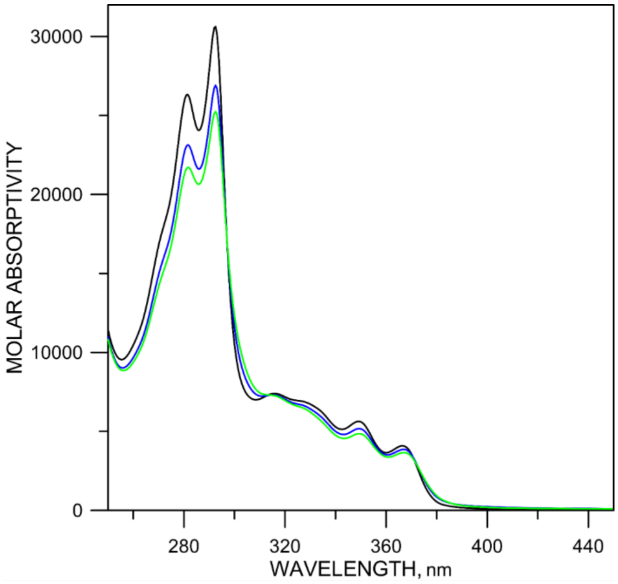

Figure S4. Absorption spectra of **6** in CH_3CN as a function of the concentration while keeping the product between the concentration value and the path length constant (5.0×10^{-5}M). The concentrations are as follows - black solid line: 1.0×10^{-4} M (most concentrated); blue solid line: 5.0×10^{-5} M and green solid line: 5.0×10^{-6} M (most diluted). At concentration higher than 1.0×10^{-4} M no changes are observed.

Table S2. Predicted absorption spectra of the monomer and dimer of **6** in DMSO.

form	*B3LYP/6-311+G(2d,p)// M06-2X/TZVP*	
	λ_{max}, nm	*f**
monomer	*344*	*0.086*
	321	*0.037*
dimer	*343*	*0.092*
	336	*0.092*
	323	*0.010*
	321	*0.023*

** Oscillator strength.*

^{1}H-NMR, ^{13}C-NMR Spectra

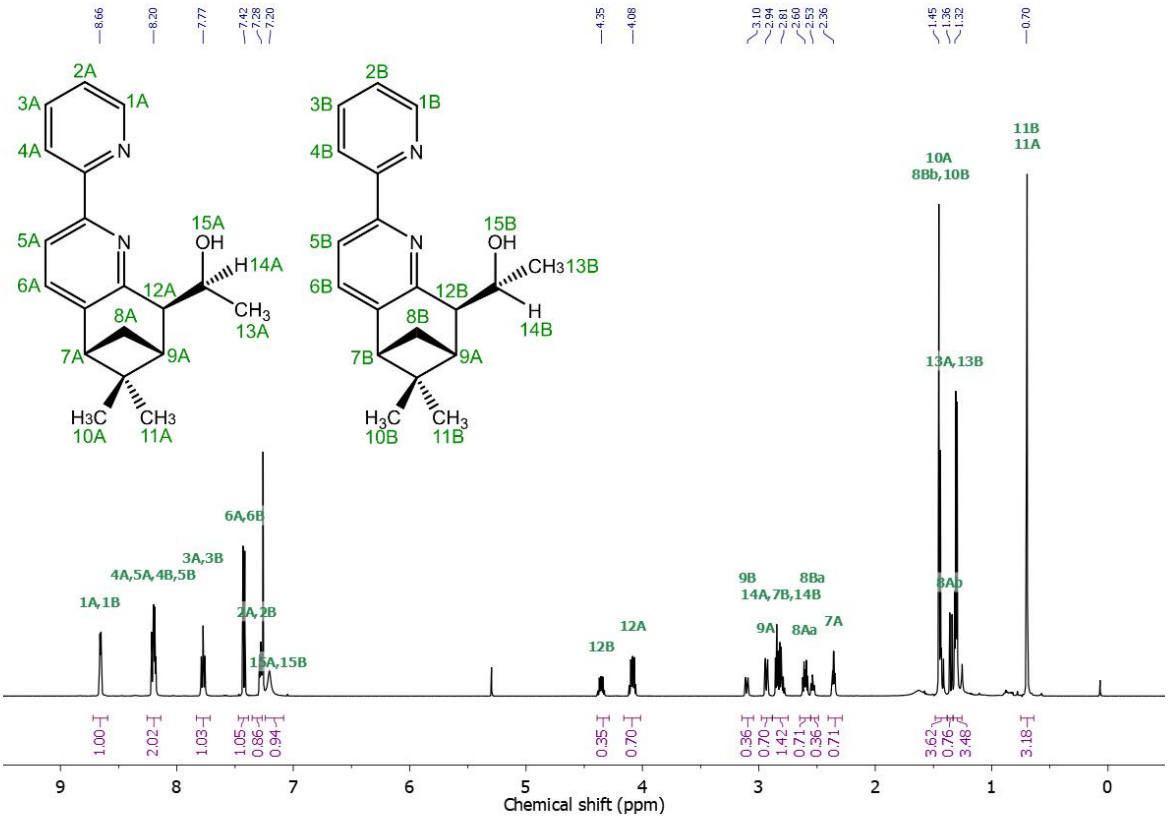

Figure S5. ^{1}H-NMR spectrum of **2** in $CDCl_3$ and proton numbering

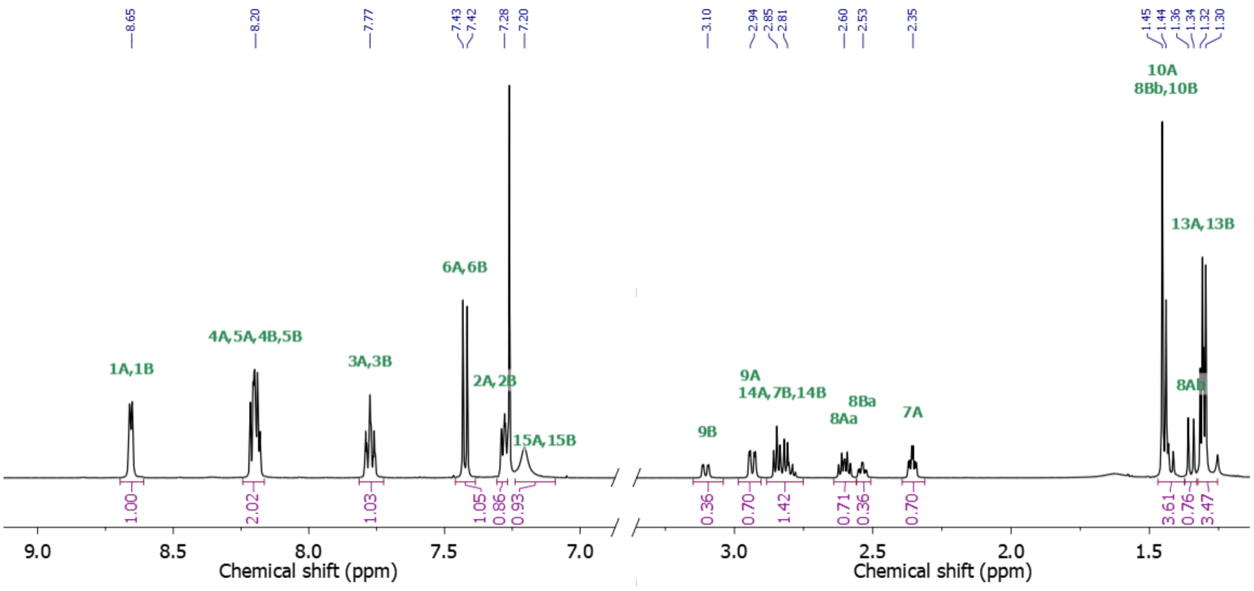

Figure S6. Zoom on the aromatic (left) and aliphatic (right) region of the ^{1}H-NMR spectrum of **2** in $CDCl_3$

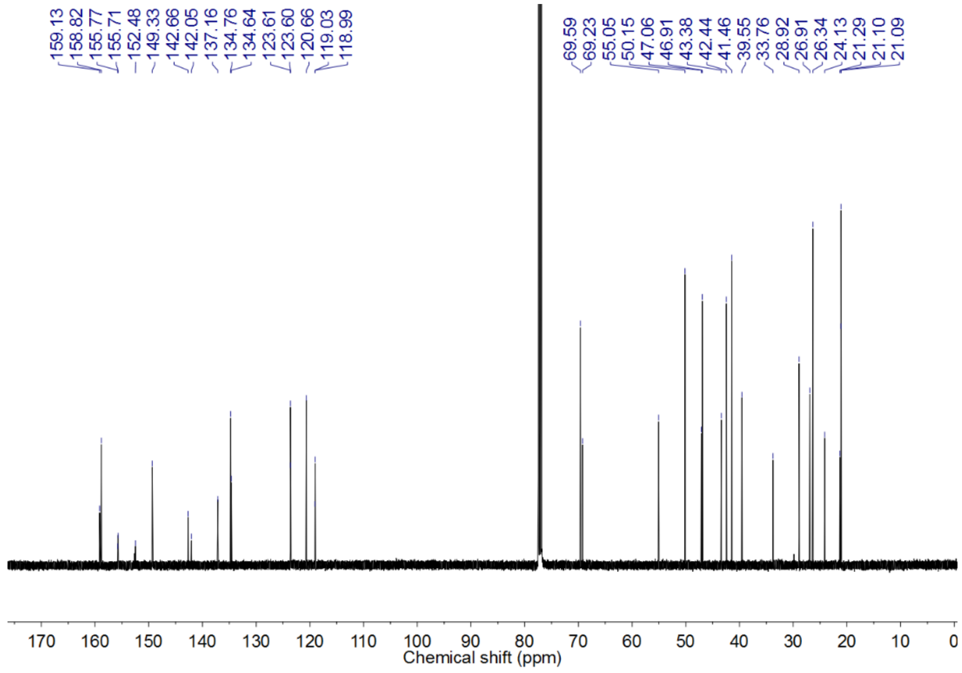

Figure S7. ^{13}C-NMR spectrum of **2** in $CDCl_3$

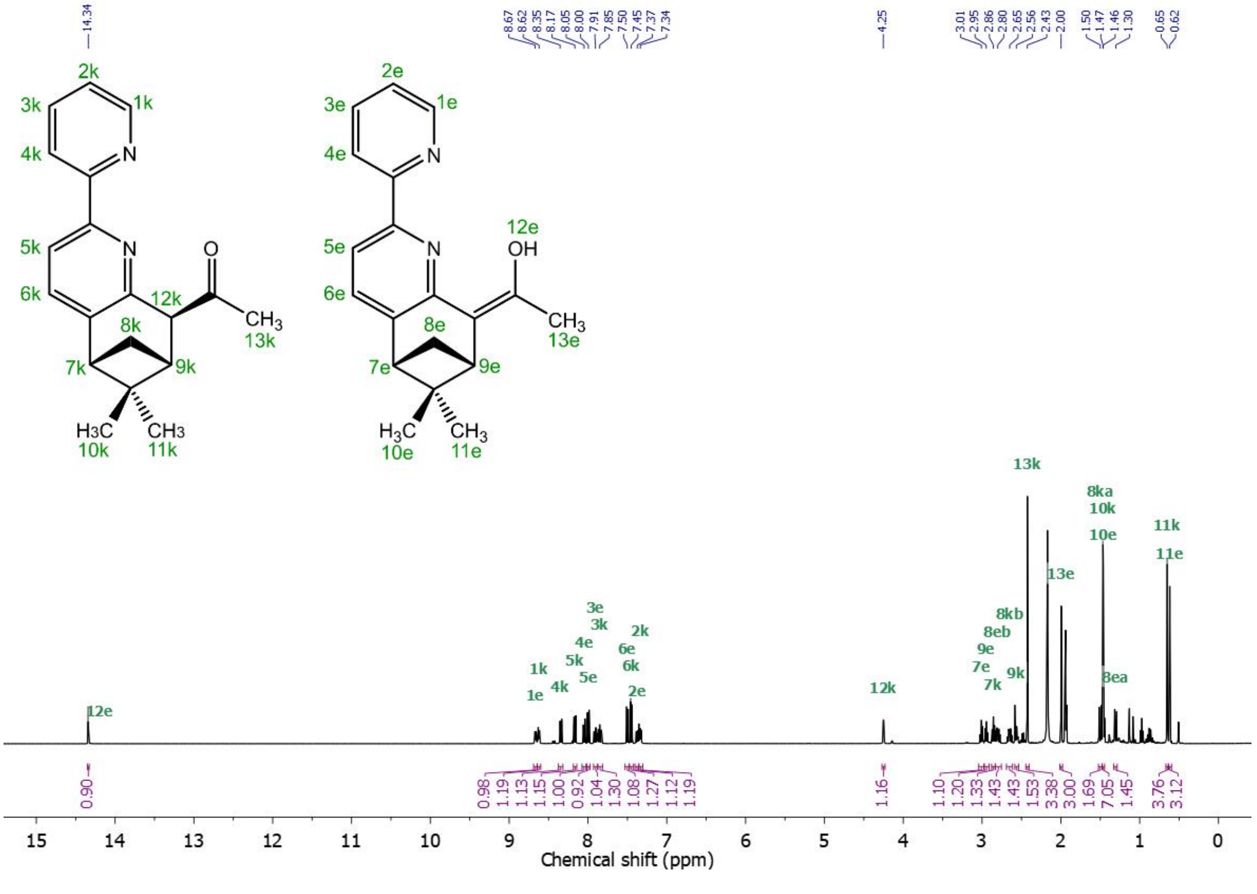

Figure S8. ^{1}H-NMR spectrum of **3** in CD_3CN and proton numbering

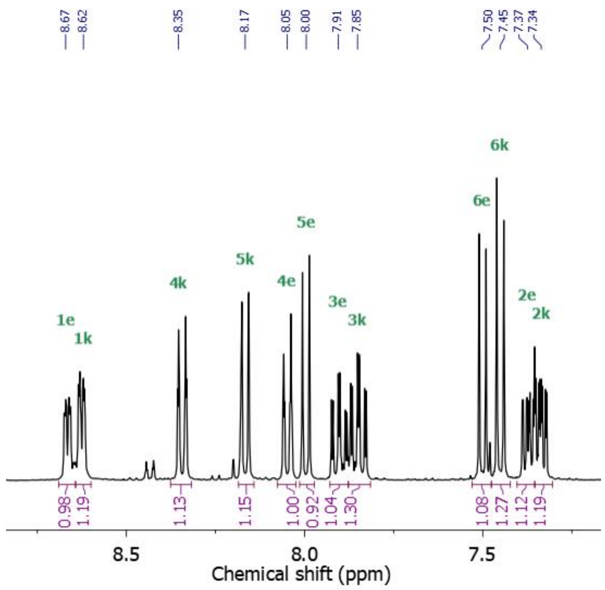

Figure S9. Zoom on the aromatic region of the ^{1}H-NMR spectrum of **3** in CD_3CN

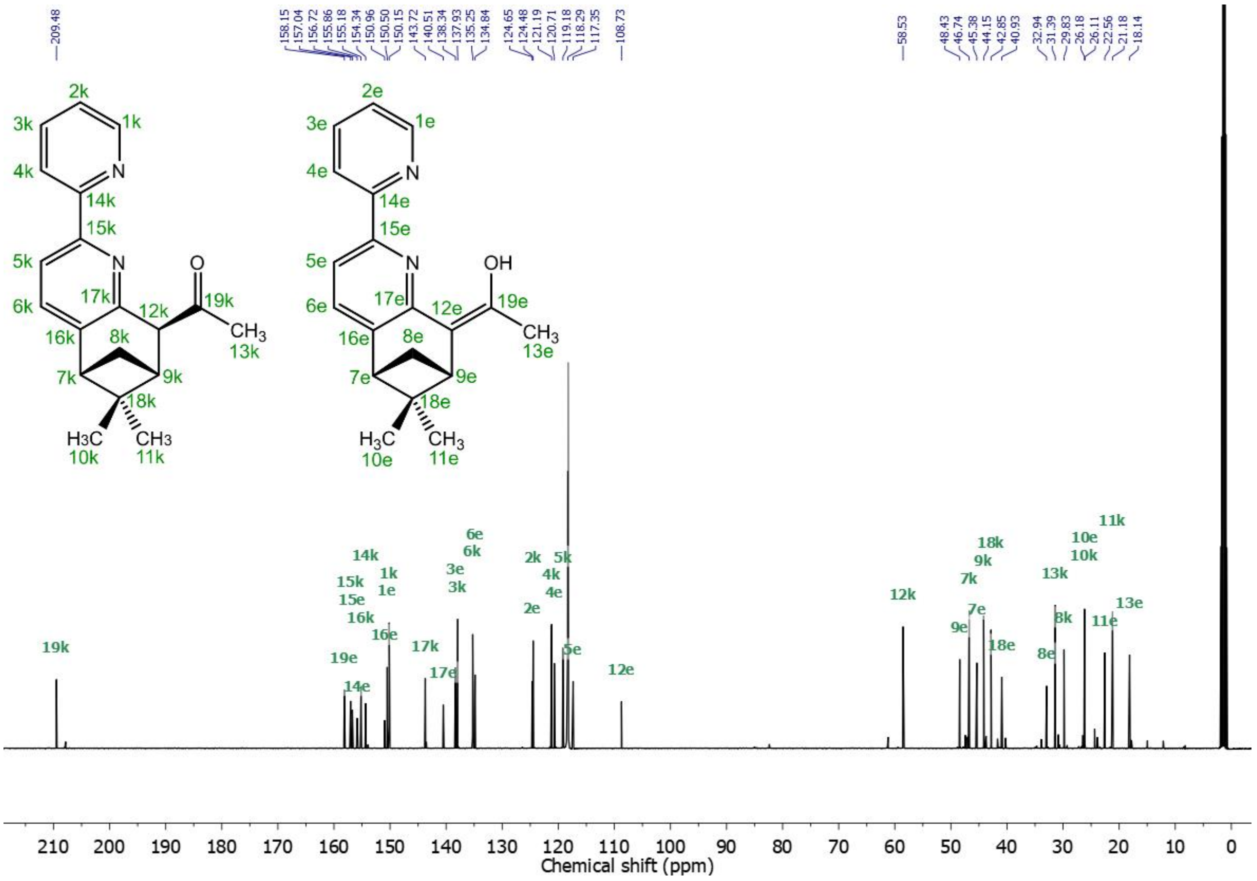

Figure S10. ^{13}C-NMR spectrum of **3** in CD_3CN and carbon numbering

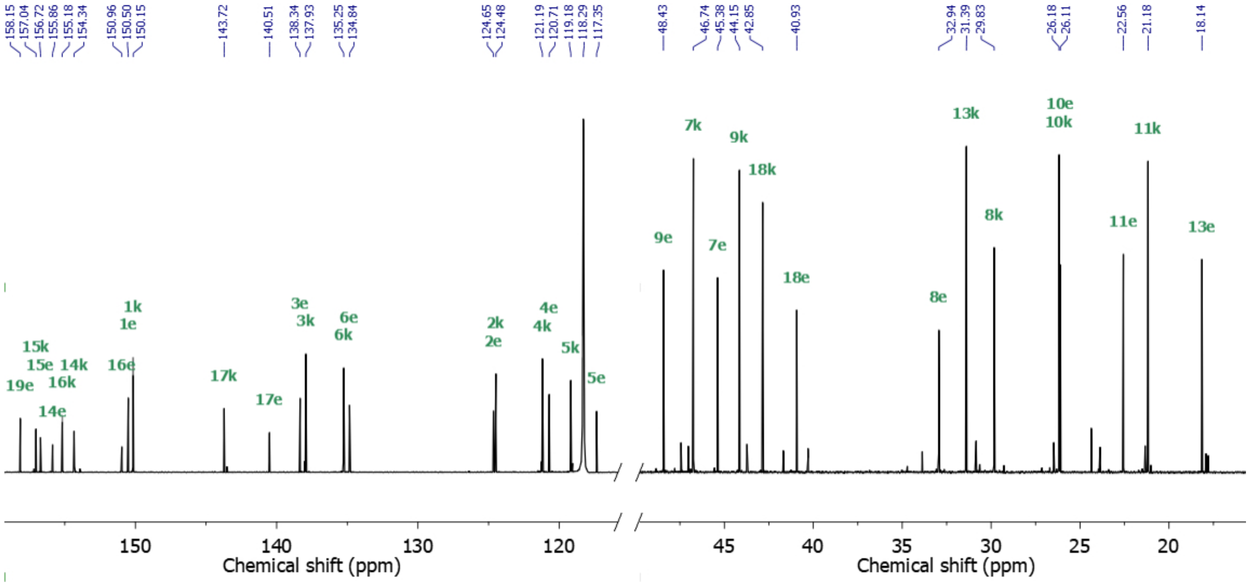

Figure S11. Zoom on the aromatic (left) and aliphatic (right) region of the ^{13}C-NMR spectrum of **3** in CD_3CN

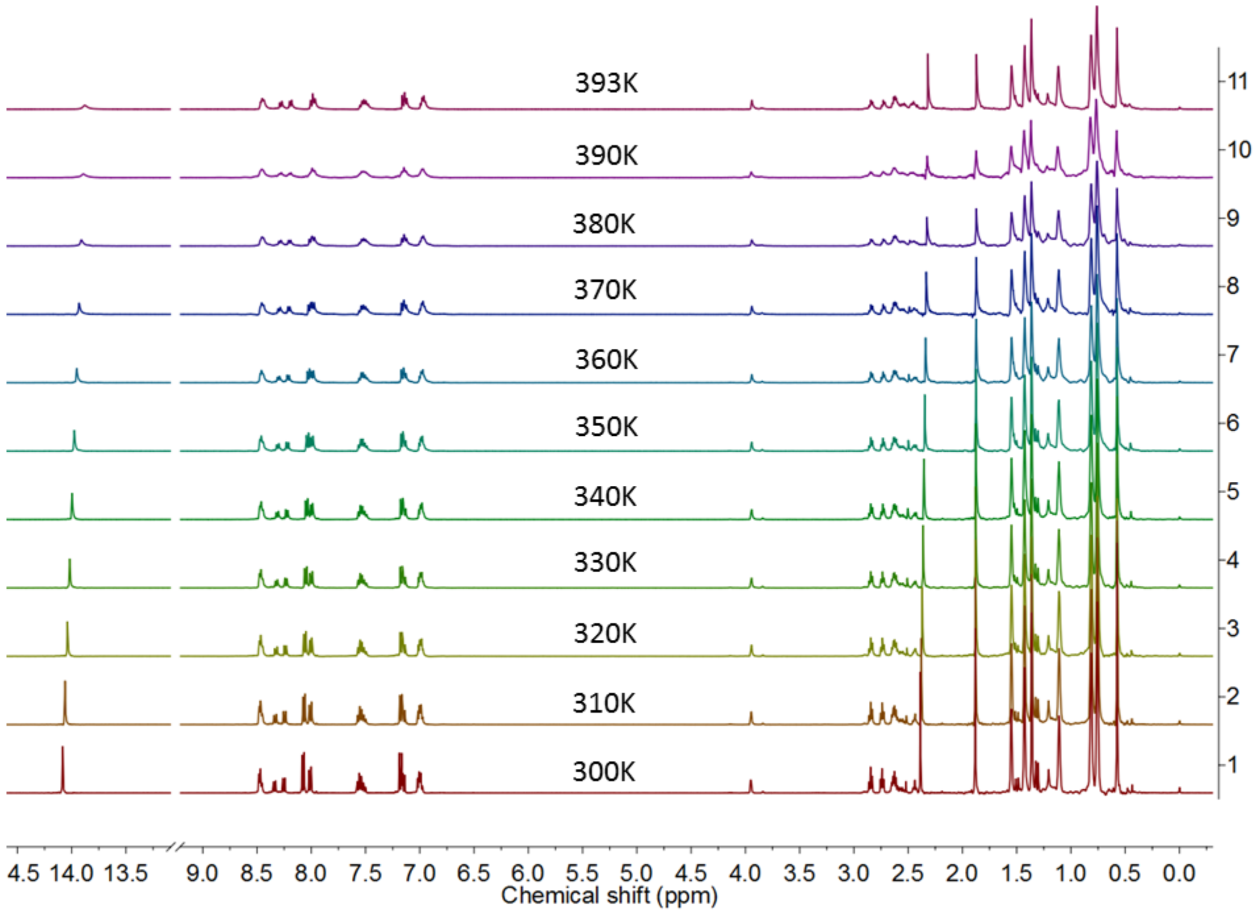

Figure S12. VT-^{1}H-NMR spectra of **3** in decalin-d18

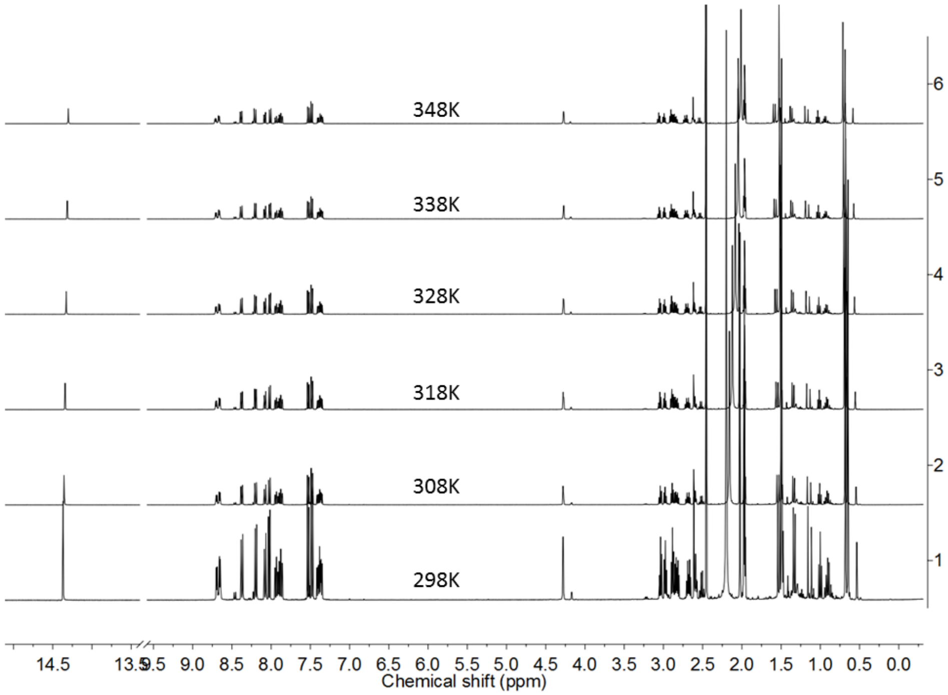

Figure S13. VT-^{1}H-NMR spectra of **3** in CD_3CN

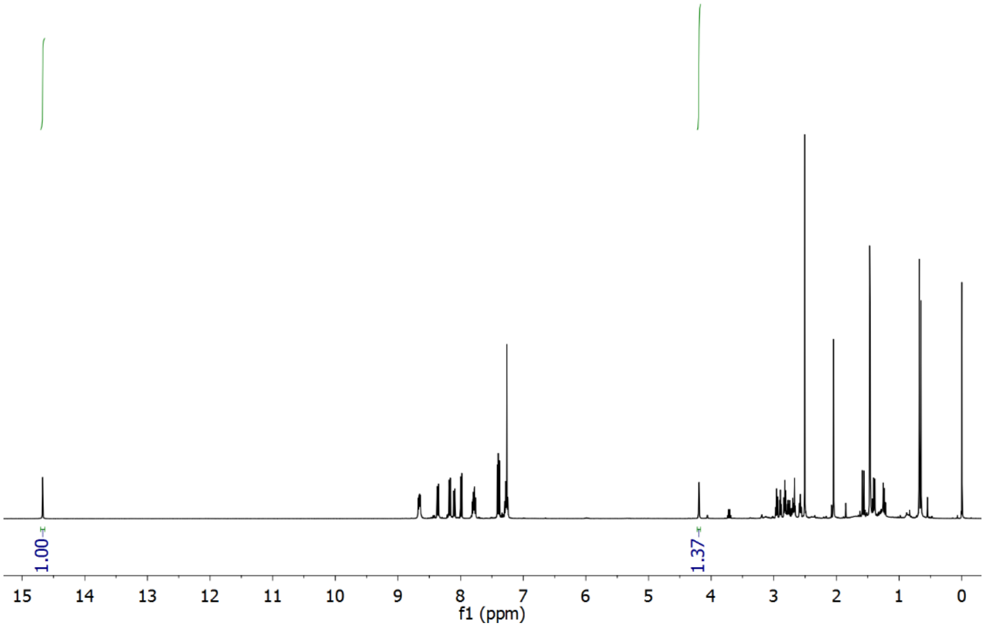

Figure S14a. ^{1}H-NMR spectrum of **3** in CD_3Cl at 298K

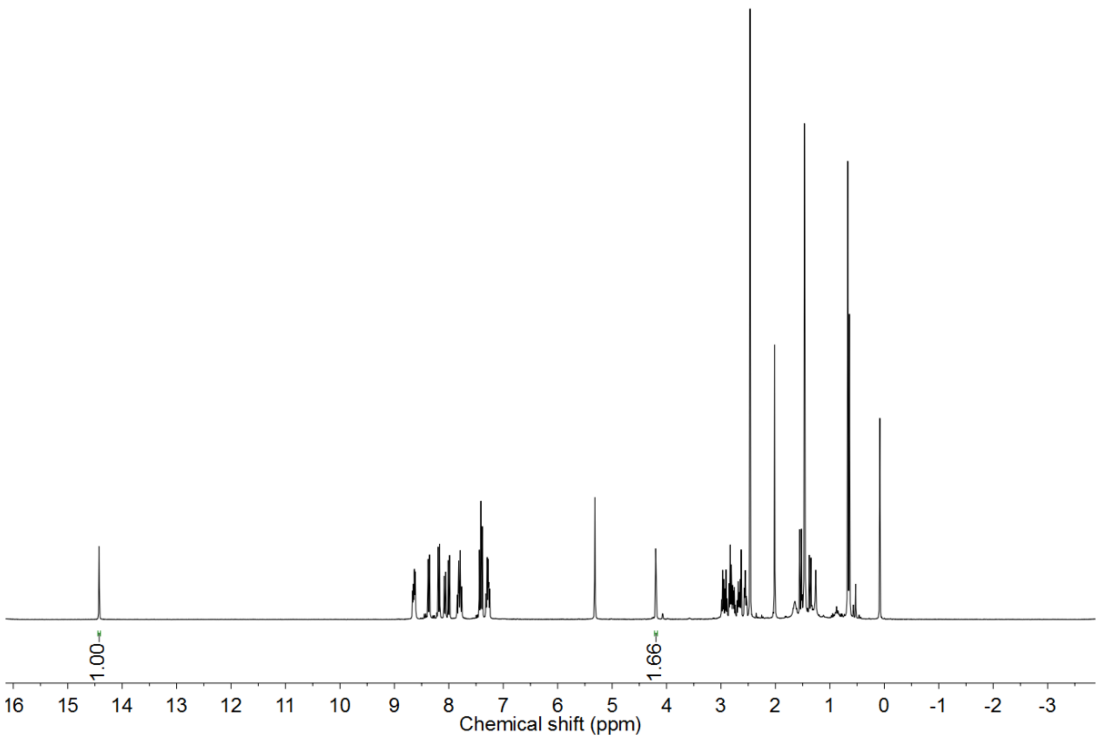

Figure S14b. ^{1}H-NMR spectrum of **3** in CD_2Cl_2 at 298K

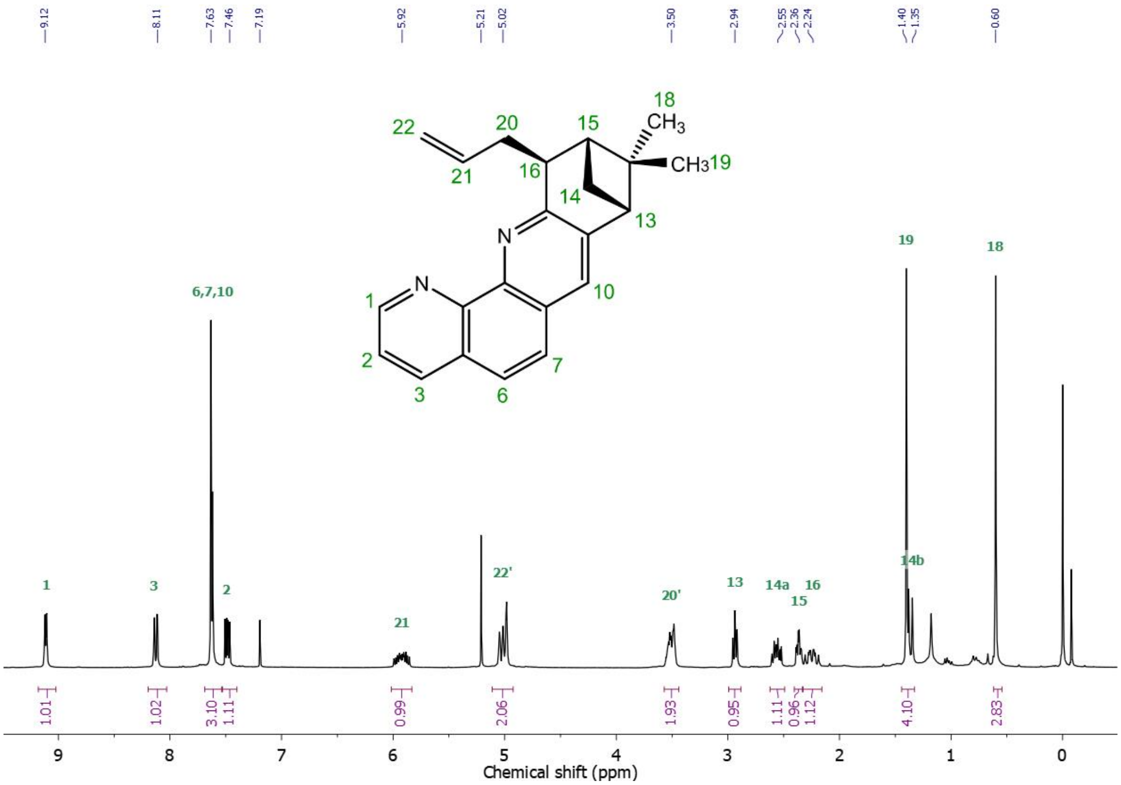

Figure S15. ^{1}H-NMR spectrum of **5** in $CDCl_3$ and proton numbering

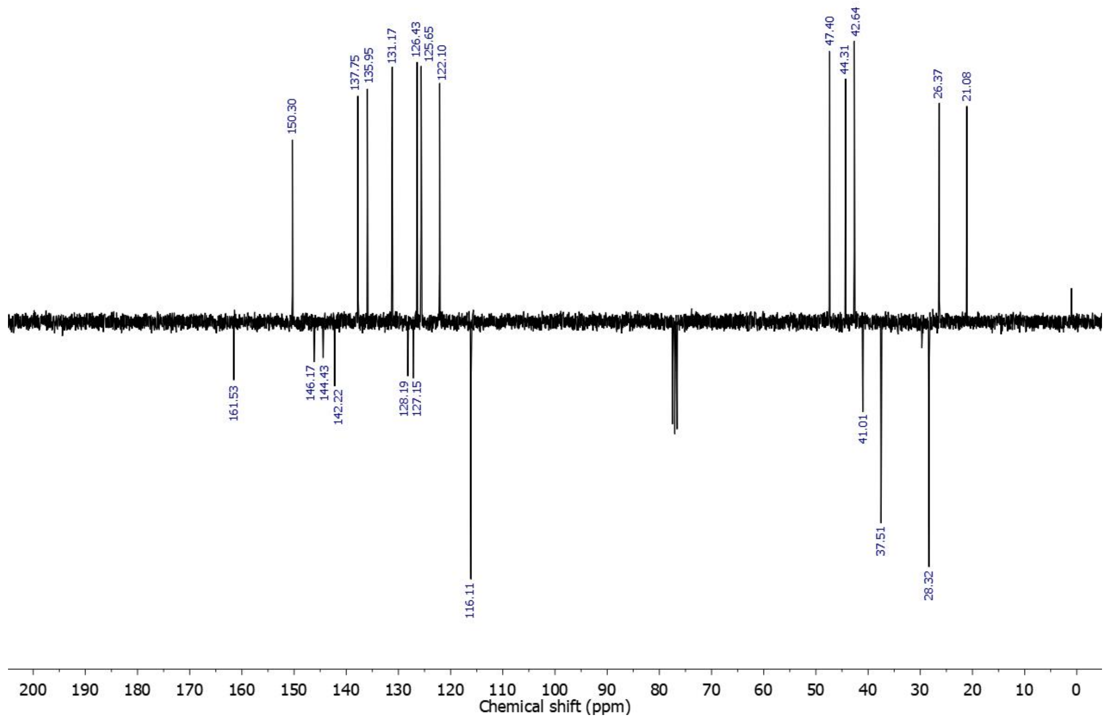

Figure S16. C-APT-NMR spectrum of **5** in $CDCl_3$

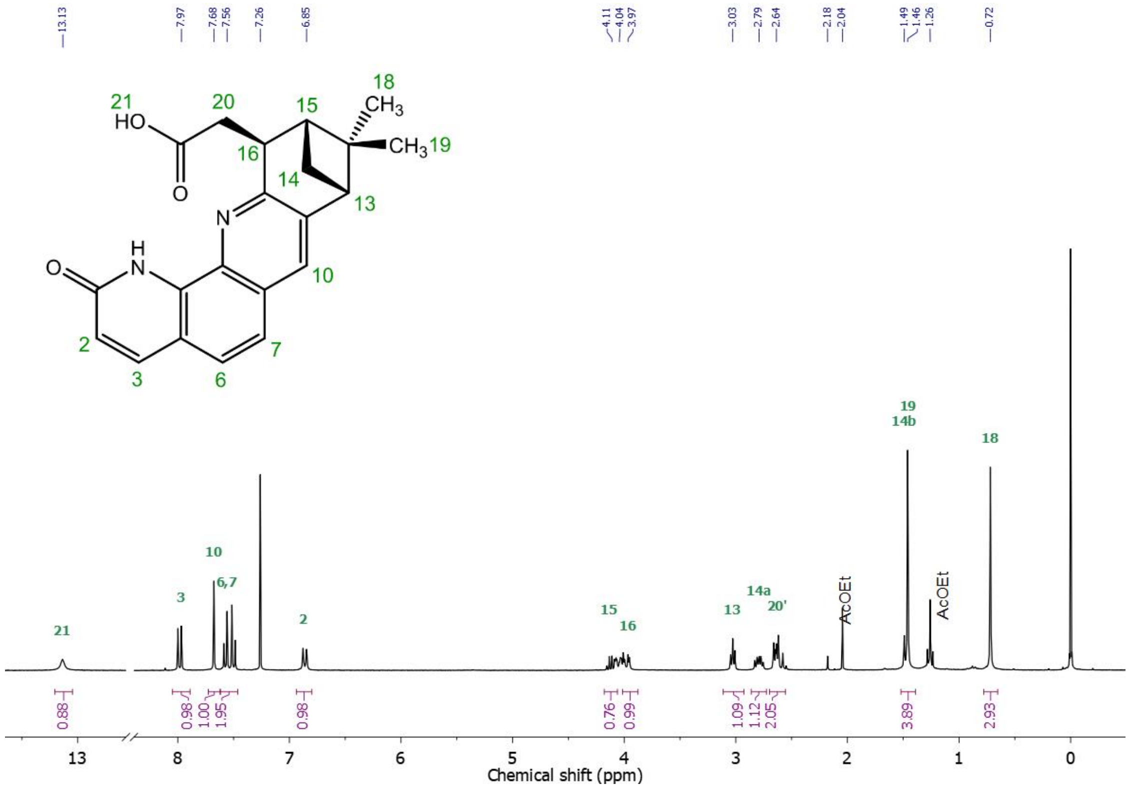

Figure S17. ^{1}H-NMR spectrum of **6** in $CDCl_3$ and proton numbering

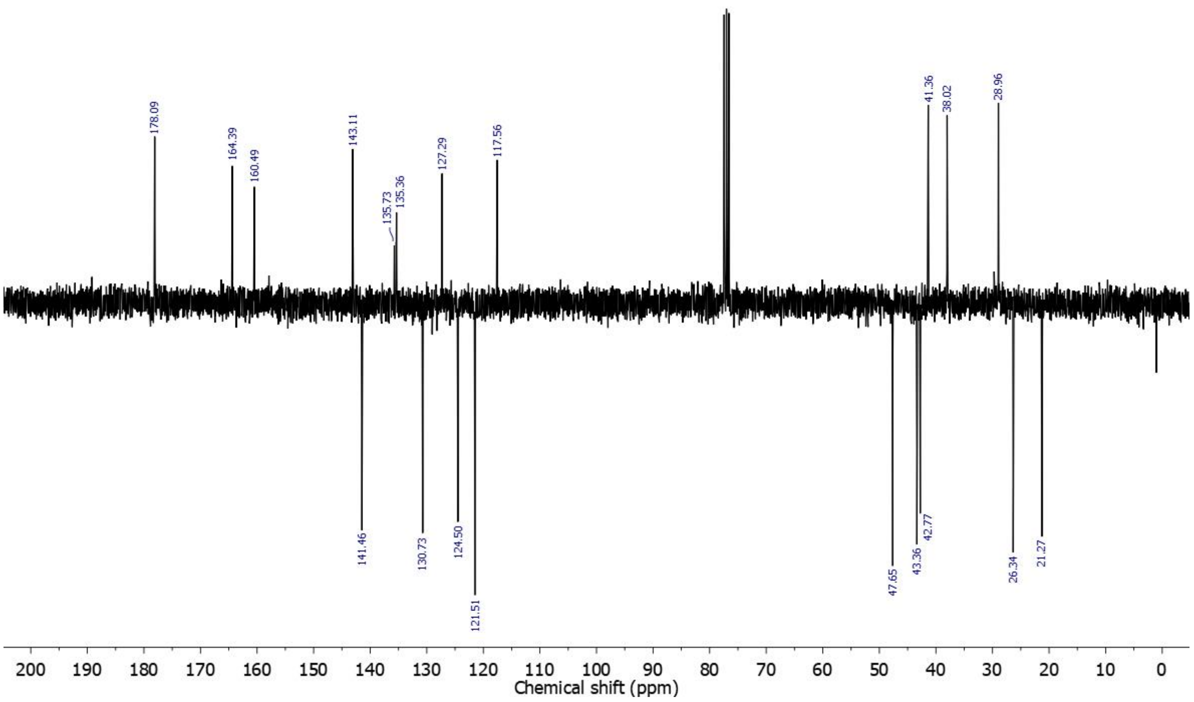

Figure S18. C-APT-NMR spectrum of **6** in $CDCl_3$

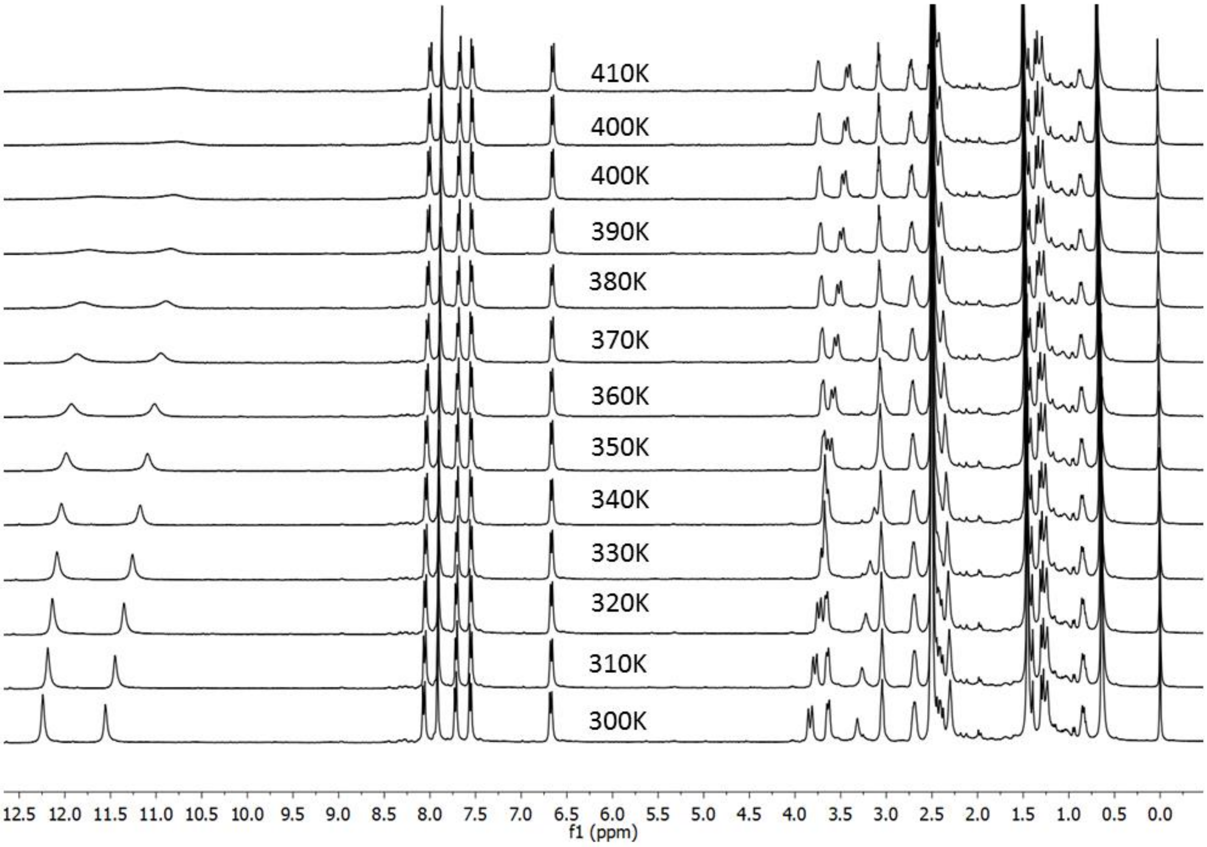

Figure S19. VT-NMR spectra of **6** in DMSO-d6

Mass spectra

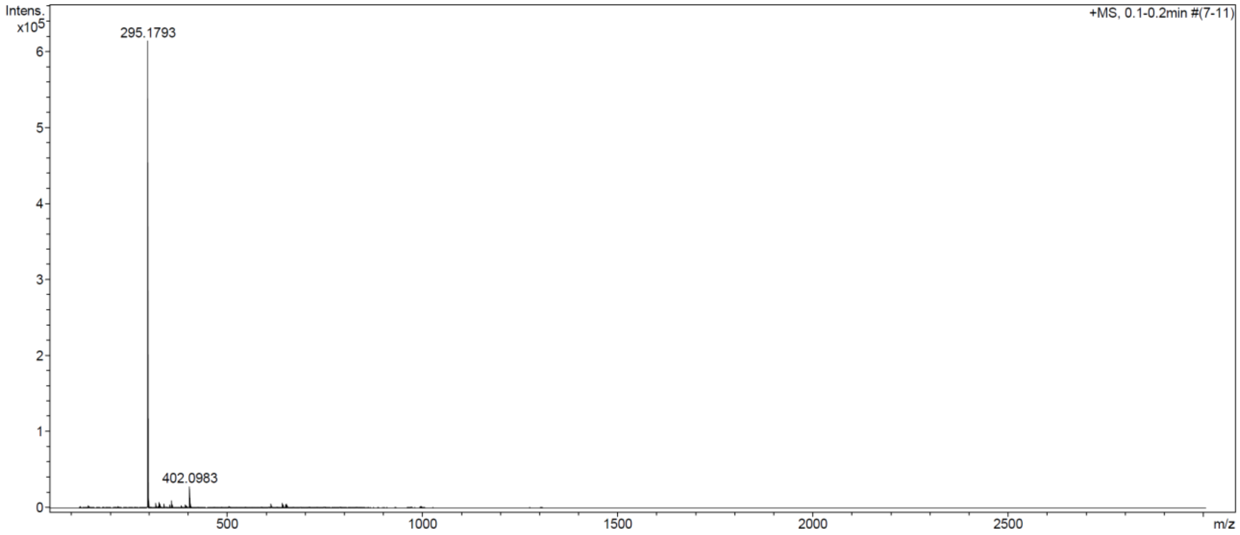

Figure S20. (+)-ESI-MS spectrum of **2**

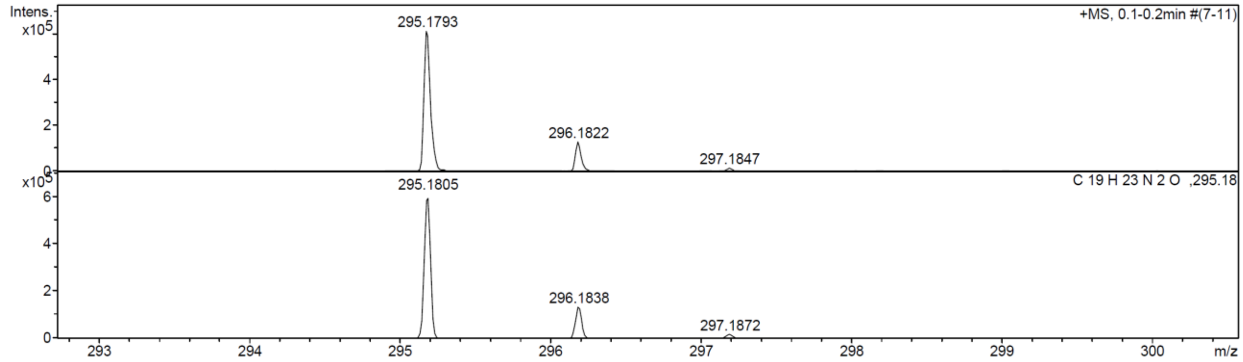

leas. m/z	#	Formula	m/z	err [ppm]	Mea n err [ppm]	rdb	N-R ule	e⁻ Conf	mSig ma	Std I	Std Mean m/z	Std I VarNor m	Std m/z Diff	Std Comb Dev
295.1793														
	1	C 19 H 23 N 2 O	295.1805	3.9	4.2	9.5	ok	even	2.50	0.0039	0.0013	0.0016	0.0004	0.8427

Figure S21. HRMS spectrum of **2**

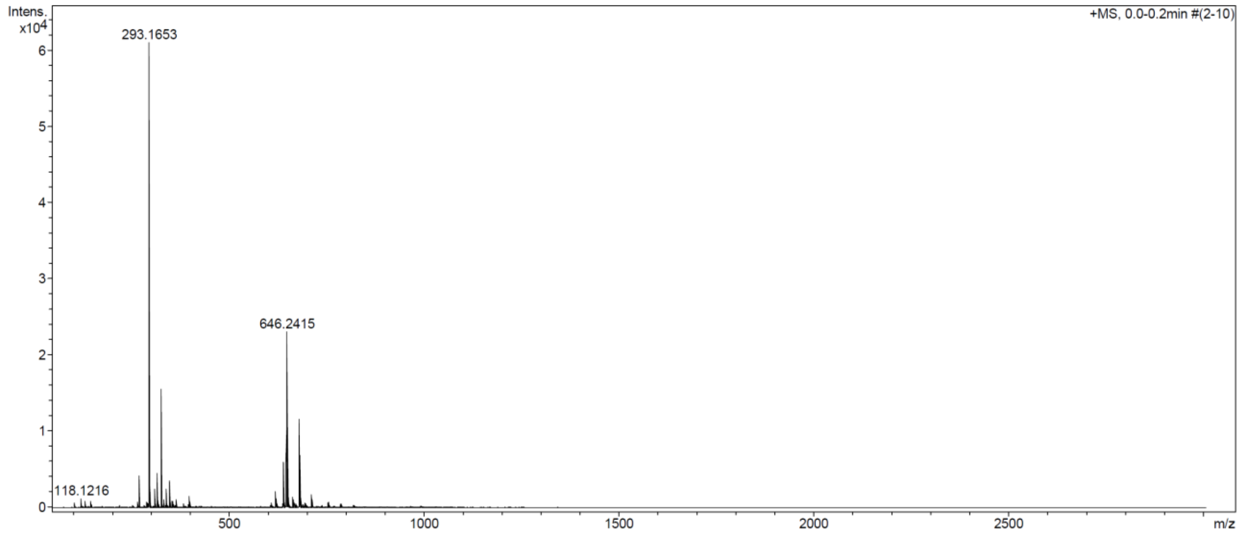

Figure 22. (+)-ESI-MS spectrum of **3**

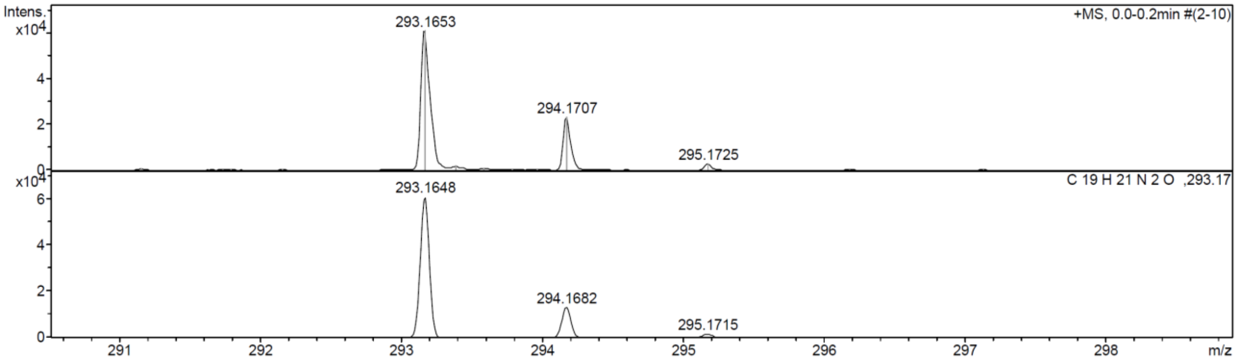

eas. m/z	#	Formula	m/z	err [ppm]	Mean err [ppm]	rdb	N-Rule	e⁻ Conf	mSigma	Std I	Std Mean m/z	Std I VarNorm	Std m/z Diff	Std Comb Dev
293.1653														
	1	C 19 H 21 N 2 O	293.1648	-1.4	-3.4	10.5	ok	even	97.29	0.1442	0.0014	0.0569	0.0021	0.8427

Figure S23. HRMS spectrum of **3**

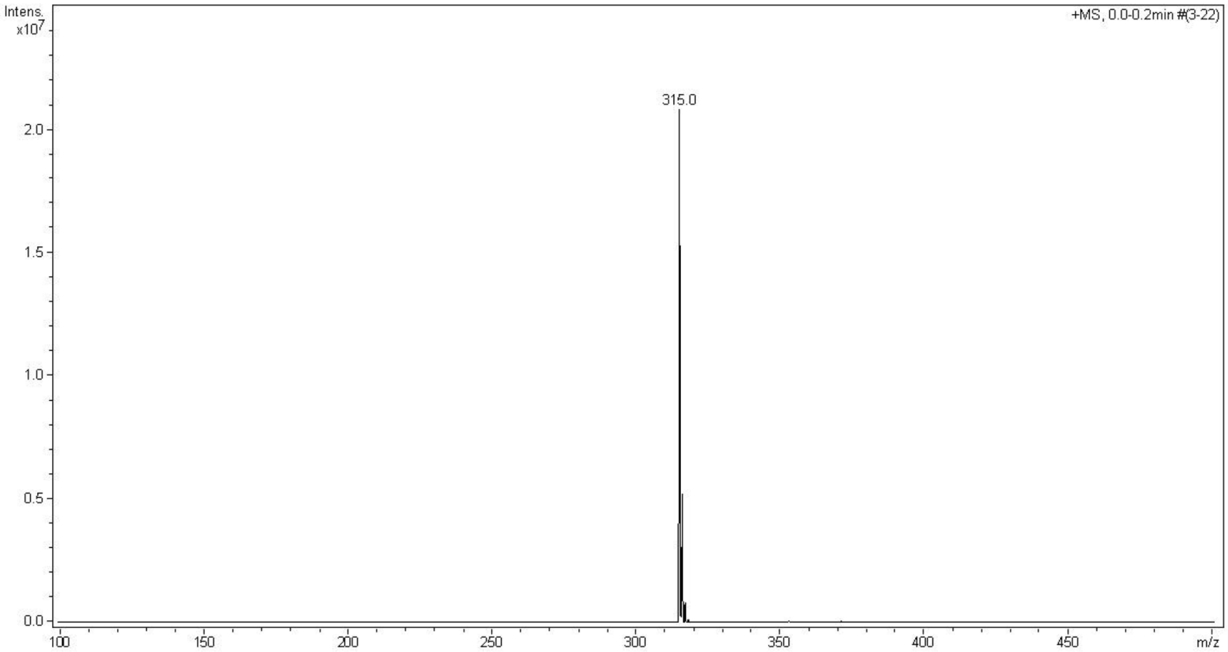

Figure S24. (+)-ESI-MS spectrum of **5**

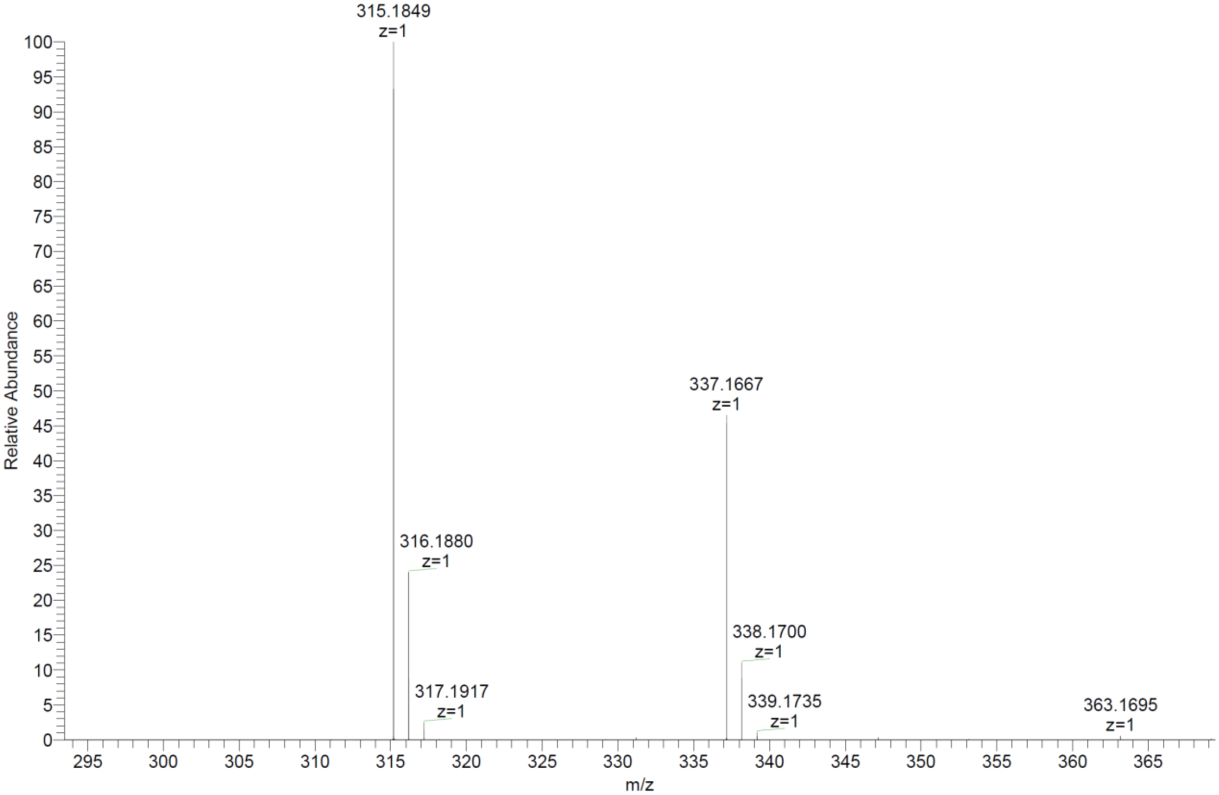

Figure S25. HRMS spectrum of **5**

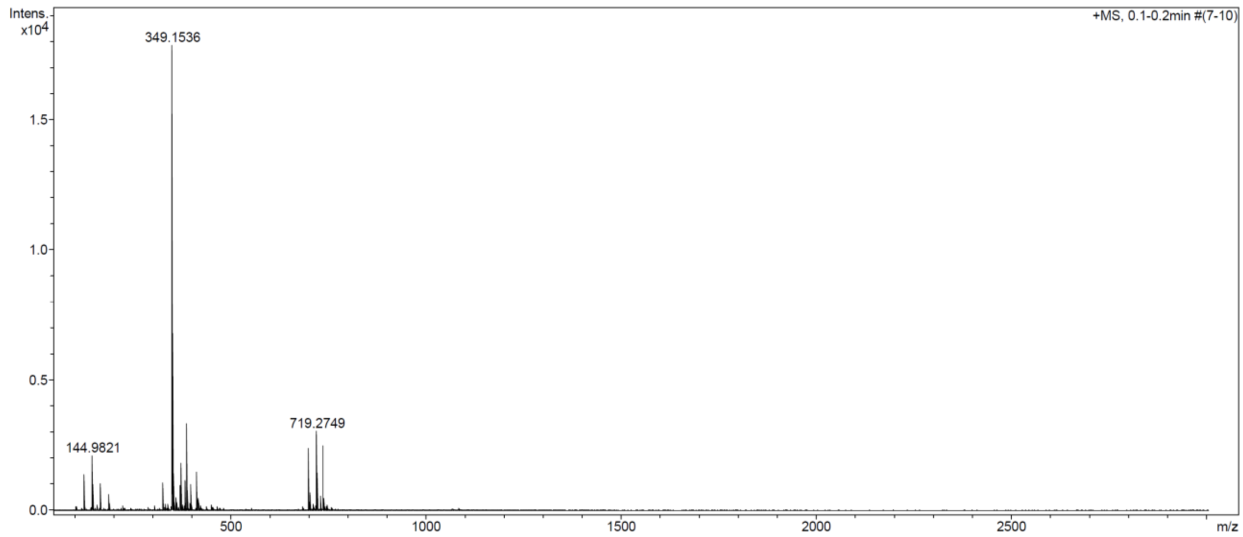

Figure S26. (+)-ESI-MS spectrum of **6**

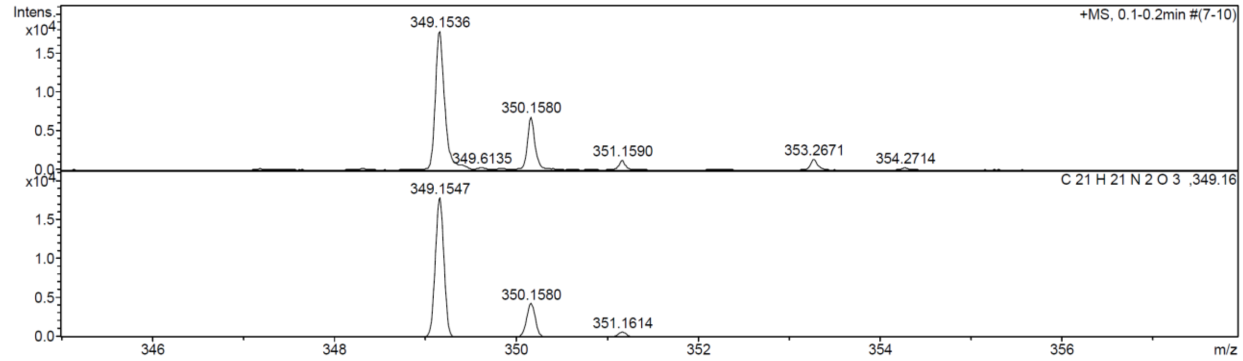

eas. m/z	#	Formula	m/z	err [ppm]	Mean err [ppm]	rdb	N-Rule	e⁻ Conf	mSigma	Std I	Std Mean m/z	Std I VarNorm	Std m/z Diff	Std Comb Dev
349.1536														
	1	C 21 H 21 N 2 O 3	349.1547	3.1	2.4	12.5	ok	even	87.77	0.1295	0.0010	0.0546	0.0011	0.8427

Figure S27. HRMS spectrum of **6**

Table S3. Crystallographic data for **6**

**Compound**	**6**
CCDC number	1958303
Formula	$C_{21}H_{20}N_2O_3$
$D_{calc.}$/ g cm^{-3}	1.324
μ/mm^{-1}	0.089
Formula Weight	348.39
Colour	colourless
Shape	needle
Size/mm^3	0.32×0.14×0.02
T/K	250(2)
Crystal System	orthorhombic
Space Group	$P2_12_12_1$
a/Å	6.2818(8)
b/Å	14.5974(17)
c/Å	19.054(3)
V/Å^3	1747.2(4)
Z	4
Z'	1
Wavelength/Å	0.71073
Radiation type	MoK$_{\alpha}$
Θ_{min}- Θ_{max}/°	1.757 - 26.365
Measured Refl.	25010
Independent Refl. / restraints/parameters	3517 / 36 /239
Reflections with I > 2(I)	878
R_{int}	0.2809
Largest Peak / Hole /e Å^{-3}	0.141 / -0.173
Deepest Hole	-0.173
GooF	0.675
R_1, wR_2 (I>=2σ (I))	0.2324, 0.0936
R_1, wR_2	0.0462, 0.0625
